# Alleviating behavioral biases at job search: Do nudges work?

**DOI:** 10.1371/journal.pone.0266105

**Published:** 2022-04-06

**Authors:** Gergely Horvath

**Affiliations:** Division of Social Sciences, Duke Kunshan University, Suzhou, China; Universidad de Alicante, SPAIN

## Abstract

We experimentally study the effectiveness of policy interventions in reducing the negative welfare effects of behavioral biases on job search. Due to quasi-hyperbolic discounting, individuals reduce their search effort and reservation wage, while the sunk-cost fallacy makes individuals decrease their reservation wage over the search spell. We compare the effects of search cost reduction and nudging. We find that search cost reduction increases the search effort and payoffs but not the reservation wage. Conversely, nudging increases the reservation wage, but not the search effort or payoffs. Both interventions reduce the impact of the sunk-cost fallacy on the reservation wage.

## 1. Introduction

Active labor market policies (ALMPs) assist unemployed workers in finding suitable new employment in a short period of time. They comprise job search assistance, training programs, subsidized public sector employment programs, and information provision on the state of the labor market and on available governmental programs (see e.g., [1–5]). Recent field experiments extend the scope of such programs by addressing the motivational problems present in long-term job seeking (see [6, 7]), and by giving advice on alternative occupations that fit the skills of the job seeker (see [8]). However, ALMPs pay remarkably little attention to addressing the behavioral biases that are present at job search. Behavioral factors that influence the job search process include the locus of control (see [9, 10]), the sunk-cost fallacy (see [11]), quasi-hyperbolic discounting and present bias (see [12, 13]), biased beliefs on the re-employment probability (see [14, 15]), social comparisons (see [16]), and reference-dependent utility (see [17]). While these behavioral factors divert the job search strategies of unemployed workers from the payoff-maximizing choices, most ALMPs do not include components that directly target them with the aim to reduce their effects on the job search process.

To fill the mentioned gap, in this paper, we analyze the impact of policy interventions that aim to address the negative welfare effects of behavioral factors on job search. We present the results of an online experiment whereby individuals participate in eight search rounds, each consisting of a stationary, infinite-horizon search task. The infinite horizon is created by the random termination method (see [18, 19]) whereby the search round continues to the next period with 95% probability. Individuals start the round as unemployed and searching for a suitable wage offer by deciding on a search effort level and a reservation wage. The search effort is equal to the probability of receiving an offer, a higher search effort is more costly according to a cost function. Search costs are paid out of an endowment. The reservation wage is the minimum acceptable offer that individuals submit before receiving an offer. Once these decisions are made, the computer randomly determines whether the individual will receive an offer, and if yes, the wage offer is randomly drawn from a known uniform distribution. If an offer is accepted, the individual receives the value of the offer at each period until the end of the search round. Otherwise, she searches again, provided that the search round continues to the next period. The payoff of the participant is equal to the sum of payoffs earned in all periods of a randomly chosen round, out of the eight search rounds of the experiment.

In our *Baseline* treatment, we observe two types of deviations from the optimal search strategies which we attribute to behavioral factors. Firstly, individuals choose a lower search effort level and a lower reservation wage than the optimal values of a rational individual discounting future payoffs exponentially. Following [12], we introduce quasi-hyperbolic discounting to the search model, and show that the mentioned deviations can be partially (but not fully) explained by quasi-hyperbolic discounting and present bias. Secondly, individuals increase the investment in search effort and decrease the reservation wage over time during the search spell. In contrast, the optimal strategy in an infinite-horizon stationary search environment is to keep these choice variables constant over time. In a theoretical model, we show that these deviations can be explained by the sunk-cost fallacy (see also [20, 21]), that is, individuals perceive that the search costs accumulate over time. We also show that both quasi-hyperbolic discounting and the sunk-cost fallacy reduce individual welfare, which motivates our policy interventions.

Turning to the proposed policy interventions, we study the impact of nudging messages displayed on the decision screen of the experiment to correct individual behavior and improve welfare. These messages warn individuals about the behavioral biases and suggest a course of action that improves their payoffs. Individuals are completely free to follow these suggestions or not. In a 2x2 experimental design, we introduce two types of nudges, *Nudge1* and *Nudge2*, and their interactions labelled as *Nudge1+2*, and compare these treatments to the *Baseline* described above. *Nudge1* intends to increase the search effort and the reservation wage, which would lead to higher welfare for the individual. *Nudge2* aims to alleviate the impact of the sunk-cost fallacy on the choice of reservation wage over time. Our nudging interventions are motivated by the recent evidence on successful implementation of similar nudges in many domains, including health, education, environmental protection, savings, and consumption behavior, etc. (see e.g., [22–24]). Warning messages and information provision are among the most important and effective nudges (see e.g., [25]). We propose that similar nudges could be added to ALMPs with the specific intention to alleviate the consequences of behavioral biases.

As a comparison, we study in an additional treatment a second type of policy intervention, a reduction in the search costs paid by individuals. Job search assistance is a standard part of ALMPs, which reduces the search costs of the unemployed worker. Lower search costs will induce a larger search effort and reservation wage leading to higher welfare. They may also alleviate the impact of the sunk-cost fallacy since individuals will perceive lower costs accumulating over the search spell. We compare the effectiveness of this standard intervention to that of the proposed nudging messages.

Turning to the results on the impact of policy interventions, we firstly evaluate whether our treatments can induce an increase in the level of search effort and reservation wage set by the participants. We find that reducing the search costs significantly increases the investment in search effort and the welfare of participants, as measured by the cumulative payoffs in a search round. However, it has no significant positive impact on the choice of the reservation wage. As for the impact of *Nudge1*, we obtain that the nudging message significantly increases the reservation wage, however, it has no significant effect on the investment in search effort and the payoffs of the participants. The two interventions thus complement each other. We also point out a limitation of nudging in that its positive effect may not be strong enough to increase individual welfare.

Regarding the effect of the sunk-cost fallacy, we observe in the *Baseline* treatment that individuals decrease the reservation wage over the periods of search. We obtain that reducing the search costs and nudging (in particular, the *Nudge2* message) both eliminate this tendency. Furthermore, we show in a separate treatment *Nudge1+2* that the two nudges, targeting different behaviors, can be combined without any loss in their effectiveness. Instead, the two nudges reinforce each other, increasing the reservation wage to a larger extent than a single nudge.

Our paper extends the growing nudging literature [22–24] by showing that behavioral interventions can be useful parts of policy tools in the domain of labor market policy (see the general discussion in [26, 27]). In particular, we suggest that behavioral interventions can be added to ALMPs [1–4] in order to reduce the impact of behavioral biases on job search. In addition, we contribute to the experimental literature on job search (see e.g., [11, 16, 28–33]). This literature experimentally tests comparative statics results from various forms of search models. While most of these papers concentrate on the choice of reservation wage, we also consider the choice of the search effort in a way that can be compared to the theoretical predictions. A second contribution to this literature is that we propose policy interventions to correct the declining tendency of the reservation wage over the search spell, while the previous papers only document and explain this phenomenon [11, 16, 28, 33].

We acknowledge the limitations of our study. Firstly, our findings are based on a stylized search experiment from which the findings may or may not generalize to the field. To apply our findings to the field, one necessarily has to adjust the interventions studied here, by finding an appropriate framing for our nudging messages and identifying the most cost-effective way of reducing the job search costs (which may already be part of ALMPs). We see the role of our online experiment as an easy-to-implement preliminary test of ideas which can serve as useful inputs for the design of a large-scale field experiment. Secondly, the effectiveness of our nudging messages may originate from experimenter demand effect [34]. To reduce this possibility, we emphasize in the experimental instructions that participants are free to follow or not the messages that appear on the screen. In addition, the experimenter demand effect in lab experiments partly follows from the hierarchical relationship between the experimenter and the participants, who are often university teachers and students, respectively. Our experiment was carried out online in an anonymous setting where there is no such relationship. Nevertheless, it may still be the case that participants are confused about the experimental task and mechanically follow the advice given by the experimenter. However, our results in the *Nudge1* treatment suggest that this may not be fully the case since in that treatment participants are advised to set a higher search intensity, but *Nudge1* has no significant effect on this variable.

Our paper is organized as follows. In Section 2, we introduce the experimental framework which serves as our *Baseline* treatment. We discuss two behavioral models of job search, one introducing quasi-hyperbolic discounting, the other the sunk-cost fallacy, to the standard job search model. Finally, we contrast the results of the *Baseline* treatment with the theoretical predictions from these models. In Section 3, we describe the experimental treatments introducing policy interventions to the experimental framework and discuss the results on the impact of these interventions. Section 4 concludes the paper.

## 2. Behavioral biases at job search

### 2.1. Experimental design: Baseline treatment

We start the description of the experiment with the *Baseline* treatment which we will use to compare the actual job search behavior with the theoretical predictions. The experiment consists of eight rounds, each round comprises an infinite horizon search task. Infinite horizon is created in the experiment by using the random termination method (see [18, 19]), which implies that the number of decision periods within a round is random. We assume that a round will continue to a next period with probability δ = 0.95, while the round is terminated after the current period with probability 1-δ = 0.05. Rational, forward-looking decision-makers will thus discount future payments by a per-period discount factor equal to δ = 0.95.

Participants start each round as ‘unemployed’ and searching for an offer. In every period when they search, they receive b = 30 points as an endowment and they choose a search intensity and a reservation wage. The search intensity *s* is an integer number between 1 and 100 and it is equal to the probability of the individual receiving an offer in this period. Search is costly, the cost of choosing search intensity s is given by the increasing and convex function $C\left(s\right)=5+100{\left(\frac{s}{100}\right)}^{4}$. Participants were informed about the search costs by a table that showed the cost for each possible choice of search intensity. This table appeared in the experimental instructions and on the decision screen in every period when the participant searched. Search costs are paid from the endowment of the individual; therefore, the payoff from a period of searching is equal to the endowment minus the search costs paid. Note that in a given period, payoffs can be negative if the participant chooses a high search intensity. With respect to the reservation wage *R*, it is an integer number between 1 and 100. It represents the individual’s minimum acceptable offer: she will accept any offer that is at least as high as the reservation wage and reject any offer that is below the reservation wage. The choice of reservation wage is binding, it cannot be changed after the participant has received an offer.

Once the search intensity and the reservation wage are submitted, the computer randomly determines whether the participant will receive an offer and what the value of the offer will be. Offers are randomly drawn from the discrete uniform distribution between 1 and 100 points (only integer values are considered). On the one hand, if the participant does not receive an offer or the offer is below the reservation wage, the participant searches again in the next period, provided there is a next period. If the round ends while the participant is still searching, she moves to the next round and continues searching as part of the new round. On the other hand, if the randomly drawn offer is at least as high as the reservation wage, the individual accepts the offer and her payoff in *each* remaining period of the round will be equal to the value of the offer.

Individuals are informed about the outcome of their search right after submitting their search strategies and before the next period starts. They receive feedback on whether they obtained an offer, the value of the offer, whether they accepted the offer, their chosen search intensity and reservation wage levels, the search costs paid and the points earned in the given period. The search continues until the individual accepts an offer, in which case she moves to the end of the round, or until the round is ended by the random termination mechanism. At the end of a round, participants receive feedback on the outcomes of the round: the number of periods in the round, whether they accepted an offer and, if yes, in which period they accepted it and what the value of the offer was, and the total payoff obtained in the round. The total payoff earned in a given round is the sum of payoffs from all periods of the round, which is given by the following equation:

$$\pi_{ir}=\sum_{t=1}^{T_{r}}I\left(S_{it}=0\right)\left[b-C\left(s_{it}\right)\right]+I\left(S_{it}=1\right)w_{ir}$$ (1)

where *T*_*r*_ is the number of periods in round *r*, *I*(.) is the indicator function, *S*_*it*_ is the status of individual *i* in period *t*, which may be unemployed (coded as 0) or employed (coded as 1), and *w*_*ir*_ is the value of the offer accepted by the individual in round *r* (if any).

At the end of the experiment, one out of the eight rounds is randomly chosen for payment. Points are converted to British pounds at the rate 500 points = 1 GBP. In addition to the payment obtained from the randomly chosen round, participants received a fixed show-up fee equal to £2.5.

The experiment was conducted online through the Prolific website [35] between May and September 2020. There were 157 participants in the *Baseline* treatment. We collected information on the participants demographics (age, gender, education level, country of residence, student and work statuses), elicited their risk and time preferences, and asked them to solve a cognitive reflection test. We describe the experimental procedures and sample characteristics in more detail in the S1 Appendix. The experimental procedure was approved by the University Ethics Committee of the Xi’an Jiaotong-Liverpool University (approval number: 20-03-59, written consent was obtained).

### 2.2. Theoretical background and hypotheses

In this section, we introduce the theoretical background for the experiment and show how behavioral factors impact the job search strategies. Previous experimental evidence (see e.g., [11, 16]) shows that in search tasks individuals do not follow the predictions of standard search models with exponential discounting. They set a lower reservation wage than the optimal value and decrease it over the search spell while the optimal strategy in an infinite horizon framework is to keep it constant. There is much less evidence in the literature on how the choice of search intensity deviates from the optimal value. To derive reasonable predictions for the experiment, we introduce two behavioral models of search, based on quasi-hyperbolic discounting and the sunk-cost fallacy.

#### 2.2.1. Model with quasi-hyperbolic discounting

The first model version contrasts the exponential and quasi-hyperbolic discounting models of job search. Our discussion follows the model introduced in [12] but considers a risk-averse job seeker, not a risk-neutral one as in [12]. We suppose a job seeker with constant relative-risk aversion (CRRA) utility function $u\left(c\right)=\frac{c^{1-\gamma }}{1-\gamma }$ where parameter γ captures the degree of risk-aversion. Individuals maximize the discounted expected utility: $u\left(c_{0}\right)+\beta \sum_{t=1}^{\infty }\delta^{t}u\left(c_{t}\right)$. When *β* = 1, individuals discount according to the exponential discounting model, in which the discounting factor between any two periods *t* and *t* + 1 is *δ*. For *β* < 1, we obtain the quasi-hyperbolic discounting model, where the discounting factor between today and the next period is *βδ*, while the discounting factor between any two future periods *t* > 0 and *t* + 1 is *δ* (with *βδ* < *δ*). The individual thus exhibits present bias captured by parameter *β*.

We consider a stationary search environment where jobseekers draw offers sequentially and decide immediately whether to reject or accept (see [36]). Wage offers, denoted by *w*, are randomly drawn from a given, known distribution with cdf *F(w)* and support $\left[\underset{\_}{w},\bar{w}\right]$. Previous offers cannot be called back. Once a wage offer is accepted, the worker receives the value of the offer for the rest of her life, that is, we do not consider job separations. Job seekers choose a reservation wage *R*, that is, the minimum acceptable wage offer and a search intensity *s*, which is equal to the probability of receiving an offer. The cost of searching is given by the convex function C(*s*), with *C’(s)>0* and *C”(s)>0*. Since the environment is stationary, the optimal search strategy consists of a constant reservation wage *R*, and a constant search intensity *s* (see [12, 37]).

We write down the expected discounted utility (value function) of a job seeker in period *t* = 0 as follows:

$$V_{0}\left(R,s\right)=\left[sF\left(R\right)+1-s\right]\beta \delta V_{1}\left(R,s\right)+s\left(1-F\left(R\right)\right)\frac{\beta \delta }{1-\delta }E_{F}\left[u\left(w\right)|w\geq R\right]+u\left(b-C\left(s\right)\right)$$ (2)

The first term shows that the job seeker receives the discounted value of searching in the next period *βδV*_1_(*R*, *s*) whenever she obtains an offer below the reservation wage, which happens with probability *sF*(*R*), or receives no offer at all, which occurs with probability *1-s*. Note that here the discounting factor is *βδ* as we consider the benefits of the next period from the perspective of *t* = 0. The second term shows the expected discounted utility of receiving an offer above the reservation wage. *s*(1 –*F*(*R*)) is the probability of receiving such an offer, and *E*_*F*_ [*u*(*w*)|*w* ≥ *R*] stands for the expected utility of the offer. Once the offer is accepted, the job seeker receives the value of it for the rest of her life starting from *t* = 1, the discounting factor is thus $\frac{\beta \delta }{1-\delta }=\beta \left(\delta +\delta^{2}+\cdots \right)$. The last term stands for the instantaneous value received while searching: the utility of unemployment benefits *b* net of search costs *C(s)*.

Note that *V*_0_(*R*,*s*) depends on *V*_1_(*R*, *s*), which is the expected discounted utility of searching in period *t* = 1 and takes the following form:

$$V_{1}\left(R,s\right)=\left[sF\left(R\right)+1-s\right]\delta V_{1}\left(R,s\right)+s\left(1-F\left(R\right)\right)\frac{\delta }{1-\delta }E_{F}\left[u\left(w\right)|w\geq R\right]+u\left(b-C\left(s\right)\right)$$ (3)

The only difference between the two value functions *V*_0_(*R*,*s*) and *V*_1_(*R*, *s*) is the discounting factor of future benefits which becomes *δ* in *V*_1_(*R*, *s*), since the discounting is between *t* = 1 and the subsequent periods when the individual receives the benefits of searching in *t* = 1. We note that the form of the value function is the same for all periods *t* ≥ 1 since the discounting factor remains *δ* in all those periods. For this reason, *V*_1_(*R*, *s*) appears on the right-hand side of Eq (3).

The model gives rise to a game between the individual’s current and future selves, each of whom chooses search intensity and reservations wage. The current self’s payoffs depend on the future selves’ decisions through the value functions in (2) and (3). Following [12], we focus on the Markov perfect equilibrium of the game, which implies that strategies do not depend on payoff-irrelevant factors, such as the choices taken in previous periods by previous selves. Moreover, we consider the optimal search strategy of a sophisticated quasi-hyperbolic agent who forms rational expectations about the future and thus anticipates that her future selves will be quasi-hyperbolic as well.

Based on these assumptions, we derive the optimal search intensity and reservation wage chosen. To obtain the optimal search intensity, the agent maximizes the expected discounted utility in Eq (2) with respect to *s*:

$$\frac{\partial V_{0}\left(R,s\right)}{\partial s}=\left[F\left(R^{*}\right)-1\right]\beta \delta V_{1}\left(R^{*},s^{*}\right)+\left(1-F\left(R^{*}\right)\right)\frac{\beta \delta }{1-\delta }E_{F}\left[u\left(w\right)|w\geq R^{*}\right]-u'\left(b-C\left(s^{*}\right)\right)C^{'}\left(s^{*}\right)=0$$ (4)

The job seeker invests in searching up to the point where the marginal benefits of search effort equal the marginal costs. The reservation wage is set by the indifference point between searching in the next period and accepting an offer equal to the reservation wage. By discounting the expected utility of these two options to the present, we obtain the condition:

$$\frac{\beta \delta u\left(R^{*}\right)}{1-\delta }=\beta \delta V_{1}\left(R^{*},s^{*}\right)$$ (5)

The findings in [13] show that the optimal search intensity and reservation wage levels are lower for a quasi-hyperbolic than for an exponential agent, that is, R* and s* are decreasing in *β* (see Proposition 2 in [12]). Quasi-hyperbolic agents search less because there is a conflict of interest between the individual’s current and future selves. The current self would like to delegate costly job search to future selves, since she discounts future benefits of searching more than they do. Future selves, however, will do the same as they become the current self with the time passing. This procrastination leads to a lower search intensity and a lower reservation wage being chosen.

To illustrate this finding, we contrast the optimal strategies for *β* = 1 (exponential discounting) and for *β* = 0.886 (quasi-hyperbolic discounting), where the latter is the calibrated value of *β* in [12]. Note that a recent meta-study of quasi-hyperbolic discounting [38] finds that, for monetary rewards, the average value of *β* obtained across studies is 0.82, a very close value to the one calculated for the job search context in [12]. We solve the model for the parameter values of the *Baseline* treatment of the experiment: *b* = 30, *δ* = 0.95, *γ* = 0.27, $C\left(s\right)=5+100{\left(\frac{s}{100}\right)}^{4}$, *w* ∼ *U*[1,100], where the value of risk-aversion parameter *γ* is calculated based on the risk-preference elicitation task of the experimental questionnaire. We obtain that for *β* = 1, the optimal values are s* = 56, R* = 66, while for *β* = 0.886, they are s* = 54, R* = 55. This finding motivates our first experimental hypothesis.

**Hypothesis 1a. Individuals set a lower search effort and a lower reservation wage than predicted by the exponential discounting model. These deviations can be explained by quasi-hyperbolic discounting**.

We also compare the two models in terms of welfare. We use the total payoffs earned in a round as the individual welfare measure because this can be observed in the experiment (see Eq 1). We calculate the prediction for the expected value of total payoffs using the value function in Eq (2), and substituting the utility for payoffs:

$$\pi \left(R,s\right)=\frac{s\left(1-F\left(R\right)\right)\frac{\delta }{1-\delta }E_{F}\left[w|w\geq R\right]+b-C\left(s\right)}{1-\left[sF\left(R\right)+1-s\right]\delta }$$ (6)

Under exponential discounting, the expected value of total payoffs is 1434.5, while under quasi-hyperbolic discounting, it is lower at 1397.9. We compare these predictions to the experimental data.

#### 2.2.2. Model with sunk-cost fallacy

The second type of behavioral factor that may affect job search is the sunk-cost fallacy [20, 21], that is, the tendency to perceive that search costs accumulate over periods as an individual is searching. The introduction of the sunk-cost fallacy makes the search problem non-stationary such that the optimal reservation wage and search intensity change over time as the sunk costs accumulate.

We introduce the sunk-cost fallacy to the model described in the previous section. For simplicity, we consider the exponential discounting model with *β* = 1, however, the impact of the sunk cost fallacy does not depend on the way of discounting. The value function of searching in period *t* can be expressed as follows:

$$V_{t}\left(R_{t},s_{t}\right)=\left[s_{t}F\left(R_{t}\right)+1-s_{t}\right]\delta V_{\text{t}}\left(R_{t},s_{t}\right)+s_{t}\left(1-F\left(R_{t}\right)\right)\frac{\delta }{1-\delta }E_{F}\left[u\left(w\right)|w\geq R_{t}\right]+u\left(b-C\left(s_{t}\right)\right)-u\left(\Xi_{t}\right)$$ (7)

Here we added the last term to Eq (2) which stands for the disutility of accumulated search costs up to period *t*. The accumulated search cost is naturally zero in *t* = 0 (Ξ_0_ = 0) and it increases according to the following equation for *t* > 0:

$$\Xi_{t}=\Xi_{t-1}+\alpha C\left(s_{t}\right)$$ (8)

that is, the job seeker adds *α* ≤ 1 share of the current period’s search cost to the perceived accumulated costs. Eq (7) implies that the present discounted value of searching decreases as search costs accumulate and *u*(Ξ*t*) increases.

The optimal choice of search intensity is obtained by maximizing the value function (7) with respect to *s* which leads to the following first-order condition (analogous to Eq 4):

$$\left(1-F\left(R_{t}\right)\right)\frac{\delta }{1-\delta }\left\{E_{F}\left[u\left(w\right)|w\geq R_{t}\right]-\left(1-\delta \right)V_{t}\left(R_{t},s_{t}\right)\right\}=u\text{'}\left(b-C\left(s_{t}\right)\right)C^{\text{'}}\left(s_{t}\right)$$ (9)

Here the left-hand side represents the marginal benefits of searching which depends on the difference in utility between accepting a job and searching as captured by the term *E*_*F*_ [*u*(*w*)|*w* ≥ *R*_*t*_] – (1−*δ*) *V*_*t*_ (*R*_*t*_, *s*_*t*_). This term increases as *V*_*t*_(*R*_*t*_, *s*_*t*_) becomes lower due to the accumulating search costs in (8). The marginal benefits of searching thus increase over time. In contrast, the marginal costs of searching, represented by the right-hand side of Eq (9), are independent of the accumulating search costs and thus do not change over time. In sum, the marginal benefits of search increase, while the marginal costs of searching remain the same over time, which implies that the optimal search intensity increases over the search spell.

Turning to the optimal reservation wage, it is defined by the equation $\frac{\delta u\left(R_{t}\right)}{1-\delta }=\delta V_{t}\left(R_{t},s_{t}\right)$. Since *V*_*t*_(*R*_*t*_, *s*_*t*_) decreases over time, the left-hand side should decrease as well, reducing the optimal value of *R*_*t*_ over time. On Fig A2 in the S1 Appendix, we illustrate these results by solving the model for the experimental parameter values and various levels of *α*. Based on this model, we formulate the following hypothesis for the experiment.

**Hypothesis 1b. The reservation wage decreases and the search effort increases over the search spell**.

### 2.3. Experimental results

In this section, we contrast the experimental results from the Baseline treatment with Hypotheses 1a and 1b presented in the previous two sections. We start with Hypothesis 1a which stated that individuals will set a lower reservation wage and a lower search intensity than the values predicted by the search model with exponential discounting, and quasi-hyperbolic discounting may explain these deviations. We compare the experimental data to the theoretical predictions of Section 2.2.1 using non-parametric tests, we create independent observations for the tests by taking the average of the individual data over all rounds and periods in the experiment. The results are displayed in Table 1. For further information, we plot the distribution of key outcomes in Fig A1 in the S1 Appendix.

**Table 1:** Experimental data vs. theoretical predictions.

	Search intensity	Reservation wage	Total payoffs
Experimental data: Mean (std. deviation)	47.38 (18.58)	45.42 (17.05)	1059.2 (10.2)
Theoretical predictions for exponential discounting	56	66	1434.5
Data vs. predictions, MW-test, z-score (p-value)	-4.96 (0.000)	-10.11 (0.000)	-9.68 (0.000)
Theoretical predictions for quasi-hyperbolic discounting	54	55	1397.9
Data vs. predictions, MW-test, z-score (p-value)	-3.72 (0.000)	-5.98 (0.000)	-9.19 (0.000)
Number of observations	157	157	157

Our results partially confirm Hypothesis 1a. Comparing the data to the model with exponential discounting, we find that individuals exert significantly lower search effort than the optimal value, with an average effort level of 47.38 instead of the optimal 57 (Wilcoxon signed-rank test, p-value: 0.000). They also set a lower reservation wage: the average reservation wage is 45.42 compared to the optimal 65 (Wilcoxon signed-rank test, p-value: 0.000). These deviations are costly for the individuals as the total payoff from a round, 1059.20 in the data vs. the optimal 1437.97, is significantly lower than the theoretical prediction (the p-value of the Wilcoxon signed-rank test is equal to 0.000). Considering the model with quasi-hyperbolic discounting, we again find that the experimental data is significantly different from the theoretical predictions. This model’s predictions are closer to the experimental data but the predicted values of search intensity (54), reservation wage (55) and payoffs (1397.9) are still larger than those observed in the experiment. We thus find that quasi-hyperbolic discounting can explain some part of the deviations from the predictions of the search model with exponential discounting, but cannot explain all of it. The remaining part may be explained by other behavioral factors, such as the psychological costs of searching, or by confusion from the part of the experimental participants.

We note that if confusion is a relevant factor, the observed deviations may be only temporary and individuals may be able to learn the optimal behavior over time. Each of our participants submitted 34.24 decisions in the experiment on average (eight search rounds times 4.28 decision per round). There was thus sufficient chance for learning but we find limited evidence for it. In Fig A6 in the S1 Appendix we show the trend of reservation wage and search intensity over time. We do observe a weak upward trend for the choice of search intensity but not for reservation wage. The coefficient of Round in the regression presented in Table 2 confirms this observation formally, it is statistically significant and positive for search intensity but insignificant for reservation wage. Nevertheless, the average search intensity is 41.29 in the eighth round of the experiment, a value well below the predictions of the models. Confusing and learning thus cannot fully account for the observed deviations.

**Table 2:** Changes in search behavior over the search spell in *Baseline*.

	(1)	(2)
	Reservation wage	Search effort
Period	-0.417***	0.217*
	(0.131)	(0.128)
Duration	0.418***	-0.482***
	(0.114)	(0.179)
Round	-0.157	0.493**
	(0.233)	(0.225)
Age	1.191**	0.097
	(0.490)	(0.572)
Female	-0.525	-4.092
	(2.646)	(2.632)
Cog. Refl. Test	2.887**	5.289***
	(1.149)	(1.158)
Risk (investment)	0.037	0.042
	(0.046)	(0.044)
Vocational School	11.494***	-7.601
	(4.001)	(5.655)
Bachelor’s degree	-2.837	1.838
	(2.973)	(2.864)
Master’s degree	-1.800	1.279
	(4.168)	(4.220)
Part-time work	-5.731*	1.023
	(3.348)	(3.586)
Full-time work	-6.516	0.495
	(4.061)	(3.814)
Work experience (years)	-0.021	-0.812
	(0.574)	(0.626)
Num. previous experiments	0.330***	0.057
	(0.099)	(0.120)
Student	5.758*	-2.838
	(3.280)	(3.331)
Constant	8.732	38.932***
	(11.971)	(14.075)
Chi2	52.514	66.845
p-value	0.000	0.000
R2 within	0.010	0.017
R2 between	0.169	0.287
R2 overall	0.065	0.212
Number of observations	5,429	5,429

*Note*: Random-effects regression model with standard errors clustered at the individual level (shown in parenthesis). Sample: *Baseline* treatment. Omitted categories: Male, High school graduate, Not working, Not a student. *** denotes significance at the 1% level,

** at the 5% level,

* at the 10% level.

We summarize these findings as follows.

***Result 1*. *Individuals set a lower search effort and a lower reservation wage than the optimal values predicted by the models with exponential and quasi-hyperbolic discounting*. *The predictions of the model with quasi-hyperbolic discounting are closer to the experimental data*, *partially confirming Hypothesis 1a***.

Next, we analyze how the choices of search effort and reservation wage change over the job search spell in Baseline. Based on the model with the sunk-cost fallacy, Hypothesis 1b states that the reservation wage will decline while the search intensity will increase over the search spell. We analyze this prediction by regressing these outcomes on the number of the period when the decision was submitted and individual control variables. In all regressions, we use individual random-effects regression models and we cluster the standard errors at the individual level. We control for the participant’s age, gender, education level, work and student statuses, work experience and experience with participation in experiments, risk preferences, the results of the cognitive reflection test and the number of round when the decision was submitted. We also control for the length of the search spell before finding a suitable offer (duration) because individuals with a lower search intensity will search longer, which biases the coefficient of period downward due to selection effects. Similarly, individuals with a higher reservation wage will search longer, which introduces an upward bias for the coefficient of period. The results are shown in Table 2. We provide an alternative regression specification with more control variables in the S1 Appendix (see Table A3 in the S1 Appendix).

We find that the coefficient of period is negative and statistically significant in the regression of the reservation wage (see column 1 of Table 2), and positive and (marginally) statistically significant in the regression of search effort (see column 2 of Table 2). Our experimental results thus fully confirm Hypothesis 1b and are consistent with the search model introduced in Section 2.2.2.

***Result 2*. *In the Baseline treatment*, *individuals decrease the reservation wage and increase the search effort over the search spell*, *which can be explained by the sunk-cost fallacy*. *Hypothesis 1b is fully confirmed***.

We note that in the real-world there might be further reasons besides the sunk-cost fallacy for why individuals decrease their reservation wage over time. These reasons include skill depreciation, liquidity constraints, expiring unemployment benefits and learning about the job market. Note that these factors are mostly abstracted away from our experiment in order to focus on the behavioral factors that affect job search. We expect that due to the sunk-cost fallacy, real job seekers will reduce their reservation wage to a larger extent than what can be rationally explained by the mentioned factors. The sunk-cost fallacy will thus induce suboptimal decisions in the real world similarly to our experiment. Out of the mentioned factors, learning might be the most relevant in our experiment: even if we provide full information about all relevant aspects of the search environment, participants may not fully understand it. The theoretical literature on learning in search problems shows that learning induces declining reservation wage [39] and *declining* search intensity [40] over the search spell. Learning thus cannot explain why search intensity increases over the periods of search in our experiment, an observation which is consistent with the sunk-cost fallacy.

We note that it is unlikely that the observed deviations from the optimal behavior are the results of the random termination method used to induce infinite horizon in the experiment. [11] apply a different method based on deterministic discounting of offers to induce infinite horizon in their search experiment. They obtain qualitatively the same results: the reservation wage is lower than the theoretical prediction and it declines over the search spell. They also have an alternative treatment where they apply the random termination method, and they obtain similar results as in their original treatment. Experimental studies in other contexts also show that findings obtained by the random termination method are robust to the experimental method used to induce infinite horizon in the lab (see e.g. [41, 42]).

## 3. Policy interventions

### 3.1. Motivations for policy interventions

In this section, we introduce and test different policy interventions to increase individual welfare in the search task. From a theoretical point of view, the welfare measure and the objective function of the policy-maker is the discounted expected utility of the individual as given by Eq (2). However, considering the experimental data, we measure welfare by the total payoffs earned in a round as given by Eq (1). We note that the two measures are strongly correlated with each other since utility is a monotonic transformation of payoffs.

We start by noting that both analyzed behavioral factors, quasi-hyperbolic discounting and the sunk-cost fallacy, reduce the welfare of the individuals. In the quasi-hyperbolic discounting model, individual actions do not maximize welfare because there is a conflict of interest between the individual’s current and future selves. As discussed in Section 2.2.1., the current self discounts the future benefits of searching more than the future selves, thus would like to delegate the costly search to future selves. Future selves, however, have the same incentives when they become the current self over time. This procrastination reduces search and the individual’s welfare. [12] shows that the expected discounted utility of a quasi-hyperbolic discounter could be increased if the individual searched harder, by setting a higher search intensity and reservation wage (see Proposition 1).

Turning to the welfare effects of the sunk-cost fallacy, it is clear that the sunk-cost fallacy induces suboptimal decisions and a welfare loss since individuals will not base their decisions on maximizing the forward-looking expected discounted utility but take some past costs into account. We illustrate these welfare effects numerically in the S1 Appendix (see Fig A1 in the S1 Appendix) and show that they equally hold even if we measure welfare by total payoffs earned in a round.

Based on these arguments, we introduce policy interventions in our experiment with the intention to improve individual welfare.

### 3.2. Experimental treatments and hypotheses

We consider two types of nudging messages that inform individuals about the nature and impact of the analyzed behavioral factors and suggest strategies that will improve their payoffs from the search round. Participants will see these messages on their decision screen when choosing search effort and reservation wage. We expect that, by internalizing these messages, participants will be able to reflect on their decision situation and make a better decision.

We summarize the nudging treatments in Table 3, they form a 2x2 factorial design together with the *Baseline* treatment. *Nudge1* informs individuals about the impact of present bias and suggests setting a higher search effort and a higher reservation wage. If this nudge is effective, we expect that individuals will set a higher reservation wage and a higher search effort, which will improve their payoffs. *Nudge2* addresses the impact of the sunk-cost fallacy on the reservation wage: we suggest job seekers to maintain the level of their reservation wage after an unsuccessful search period. We thus expect that, if this nudge is effective, the reservation wage will not decrease or decrease to a lesser extent over the search spell. Note that our behavioral intervention related to the sunk-cost fallacy focuses on the choice of the reservation wage and not the search effort. This is because the sunk-cost fallacy implies an increase in the search effort over the search spell, which *improves* the payoffs of the individuals when they set a lower than optimal search effort. Therefore, the impact of the sunk-cost fallacy warrants no policy intervention considering search effort. In addition, we implement a treatment where we combine *Nudge1* and *Nudge2*, we display both messages on the decision screen. We are interested in whether these two messages can be applied together or whether they overload the attention of individuals, which may decrease their effectiveness in reducing the impact of behavioral biases. These considerations lead to our experimental hypotheses regarding the impact of nudging messages.

**Table 3:** Experimental treatments.

	Nudge 1: No	Nudge 1: Yes
**Nudge 2: No**	***Baseline*:** No messages	***Nudge 1*:** “*Individuals tend to overly focus on the current costs of searching and ignore the future benefits*, *which reduces their payoffs*. *By setting a higher search effort and a higher reservation wage*, *you may increase your payoffs from the subsequent periods*.”
**Nudge 2: Yes**	***Nudge 2*:** “*After an unsuccessful search period*, *individuals tend to decrease their reservation wage*, *which reduces their payoffs*. *By maintaining a higher reservation wage*, *you may increase your payoffs from the subsequent periods*.*”*	***Nudge 1+2*:** “*Individuals tend to overly focus on the current costs of searching and ignore the future benefits*, *which reduces their payoffs*. *By setting a higher search effort and a higher reservation wage*, *you may increase your payoffs from the subsequent periods*. *After an unsuccessful search period*, *individuals tend to decrease their reservation wage*, *which reduces their payoffs*. *By maintaining a higher reservation wage*, *you may increase your payoffs from the subsequent periods*.*”*

**Hypothesis 2a. *Nudge1* will increase the reservation wage and the search effort**.
**Hypothesis 2b. *Nudge2* will eliminate the declining tendency of the reservation wage over the search spell**.

Some discussions of our nudging treatments are in order. We are aware that the nudging messages may induce experimenter demand effect (see [34]). We try to minimize this effect by stating in the experimental instructions that subjects are free to follow or not the suggestions made in the messages. In addition, much of the experimenter demand effect is created by the authority of the experimenter as a university professor when subject are students from the same university. Since this experiment was conducted online with subjects recruited worldwide, there is no such relationship between the experimenter and the subjects. In fact, Prolific keeps the study anonymous on both sides, the experimenter and the subjects thus cannot observe each other’s identity. Nevertheless, we acknowledge that the impact of experimenter demand effect cannot be excluded and it may increase the effects of our nudging treatments.

We also note that our nudges do not intend to correct the behavioral biases (or debias individuals as in [23]) but instead aim to reduce the consequences of these biases. Our messages are similar to nudges that are described as warnings or reminders by [25]. They tend to be direct regarding what individuals should do to improve the outcomes of their decisions. They are common, for example, in the health domain where reminders are often used to induce individuals to attend health check-ups (see e.g. [43–45]). For example, [43] send the following message to remind individuals to schedule an appointment at their dentist: “Investing some time in dental health prevention today decreases your risk of a painful dental disease in the future. In addition, you may avoid considerable costs of involved treatments. Please make an appointment for your next check-up.”

In addition to the nudging treatments, we also introduce a fifth treatment, labelled as LowCost, that lowers the search costs paid by the individuals. The cost function in this treatment is $C\left(s\right)=5+37.5{\left(\frac{s}{100}\right)}^{4}$, where the marginal cost of search effort is reduced relative to the Baseline. This treatment can represent any policy measure that helps the individual with job applications, including information provision on the job search methods, or the preparation of an application package, etc. Similar interventions are standard parts of active labor market policies, and we study them here to compare their impact to the effects of nudging messages introduced above. Search cost reduction will affect the behavior of individuals both under exponential and quasi-hyperbolic discounting in the same way. The lower marginal cost of searching should induce higher investment in search effort, and also increase the reservation wage since the utility of searching will go up due to the lower costs paid for searching (increasing the right-hand side of Eq 5). Under the exponential discounting model and for the experimental parameter values, the optimal search effort changes from 56 in *Baseline* to 73 in *LowCost*, the optimal reservation wage from 66 to 69. Under quasi-hyperbolic discounting, the optimal search effort increases from 54 in *Baseline* to 70 in *LowCost*, the optimal reservation wage from 55 to 57. In addition, lower search costs should also reduce the impact of the sunk-cost fallacy since individuals will accumulate lower costs during the search spell. We thus expect that individuals will not decrease the reservation wage over the search spell or will decrease it to a lesser extent than in *Baseline*. These effects of lower search costs should improve individual welfare. Based on these arguments, we formulate the following hypotheses for the experiment.

**Hypothesis 3a. *LowCost* will increase the search effort and reservation wage**.
**Hypothesis 3b. *LowCost* will reduce the impact of the sunk-cost fallacy, that is, the change in the reservation wage over the search spell**.

The number of participants in these policy treatments are as follows: 145 participants in *LowCost*, 141 in *Nudge1*, 144 in *Nudge2*, and 153 in *Nudge1+2*. Further details of the experimental procedures are provided in the S1 Appendix.

### 3.3. Experimental results I: Alleviating the impact of present bias

In this section, we study whether our experimental interventions can induce a higher search effort and a higher reservation wage. We study the treatment effects by regression analysis in order to control for a number of factors that may vary between treatments and influence the treatment comparisons. As above, we run individual random-effects regressions and cluster the standard errors at the individual level. We control for a range of demographic factors, risk preferences, and the results of the cognitive reflection test. We pool the data from all treatments, and introduce treatment dummies for each treatment, with Baseline being the omitted category in the regressions. The outcome variables are the choices of reservation wage and search intensity, and the total payoffs earned in round. The results are displayed in Table 4. Non-parametric tests comparing the other treatments to Baseline are available in Table A2 in the S1 Appendix and yield similar results to the regression analysis.

**Table 4:** Treatment effects.

	(1)	(2)	(3)
	Search effort	Res. Wage	Total payoff
*LowCost*	5.971***	1.353	53.864**
	(2.140)	(1.797)	(23.962)
*Nudge1*	3.090	4.397**	18.553
	(2.045)	(1.932)	(23.362)
*Nudge2*	-3.578*	1.844	33.725
	(1.939)	(1.855)	(25.525)
*Nudge1+2*	1.076	5.864***	6.700
	(1.967)	(1.810)	(22.021)
Period	-0.068**	0.003	66.453***
	(0.035)	(0.058)	(1.034)
Round	0.810***	-0.185*	-4.178**
	(0.109)	(0.105)	(2.073)
Age	0.042	0.658***	5.045
	(0.324)	(0.241)	(3.518)
Female	-2.254*	-0.803	-7.476
	(1.331)	(1.224)	(15.512)
Cog. Refl. Test	4.551***	1.894***	21.497***
	(0.587)	(0.524)	(7.035)
Risk (investment)	0.039	0.110***	0.356
	(0.024)	(0.022)	(0.282)
Vocational School	-0.635	2.090	14.051
	(3.062)	(2.592)	(37.837)
Bachelor’s degree	2.343	-0.856	-0.766
	(1.563)	(1.368)	(17.715)
Master’s degree	1.610	0.659	5.820
	(2.203)	(2.010)	(25.588)
Part-time work	-0.064	1.102	-5.004
	(1.765)	(1.495)	(19.089)
Full-time work	-2.272	-0.299	-19.263
	(2.016)	(1.783)	(22.787)
Work experience (years)	-0.399	-0.020	-6.236*
	(0.339)	(0.248)	(3.546)
Num. previous experiments	0.019	0.055	-0.478
	(0.085)	(0.073)	(0.866)
Student	3.302**	3.237**	-8.190
	(1.635)	(1.386)	(17.722)
Constant	33.230***	19.308***	-305.885***
	(7.702)	(6.019)	(81.740)
Chi2	201.225	84.848	5,589.023
p-value	0.000	0.000	0.000
R2 within	0.012	0.001	0.843
R2 between	0.159	0.092	0.592
R2 overall	0.108	0.053	0.824
Number of observations	25,350	25,350	5,798

*Note*: Random-effects regression model with standard errors clustered at the individual level (shown in parenthesis). Sample: all treatments. Omitted categories: *Baseline* treatment, Male, High-school graduate, Not working, not a student. *** denotes significance at the 1% level,

** at the 5% level,

* at the 10% level.

As discussed above, *LowCost* captures any intervention that reduces the cost of searching. We expect that reducing the search costs will increase the investment in search effort and increase the reservation wage (as stated in Hypothesis 3a). The results in column (1) of Table 4 indicate that *LowCost* has indeed significantly increased the search effort set by the individuals relative to *Baseline*. The coefficient of *LowCost* dummy is positive and significantly different from zero. Regarding the reservation wage, we find that the coefficient of *LowCost* dummy is positive but not statistically significant (see column 2). Finally, the *LowCost* dummy is positive and statistically significant in the regression of the total payoff from a round (see column 3). Our results thus partially confirm the predictions of Hypothesis 3a, we summarize them as follows.

***Result 3*. *Reducing the search costs leads to an increase in search effort and payoffs but has no significant effect on the reservation wage*. *Our results partially confirm Hypothesis 3a***.

Next, we turn to the analysis of the *Nudge1* treatment, which warns individuals about the negative impact of present bias and suggests that they set a higher search effort and a higher reservation wage (Hypothesis 2a). We rely on the regressions in Table 4 and focus on the coefficient of the *Nudge1* treatment dummy. This treatment dummy is positive but not statistically significant in the regression of search effort (column 1), while it is positive and statistically significant in the regression of reservation wage (column 2). Individuals seems to be willing to increase the reservation wage, perhaps because it does not lead to immediate costs to be paid, although it may increase the search duration and lead to higher costs paid later. In contrast, raising the search effort increases the costs paid in the current period and hence, it may be less attractive for the experimental participants. Our results thus only partially confirm the predictions of Hypothesis 2a. Regarding the impact on payoffs, we find that *Nudge1* has no significant effect on the payoffs of the individuals (column 3), despite increasing the reservation wage. The impact of *Nudge1* is thus not large enough to induce higher welfare.

Finally, we briefly discuss whether adding a second nudge to *Nudge1* influences its impact on the labor market outcomes. We evaluate the coefficient of the *Nudge1+2* treatment dummy in the regressions of Table 4. The most important difference between the impact of *Nudge1* and *Nudge1+2* is that the increase in the reservation wage is larger in *Nudge1+2*. This is because both *Nudge1* and *Nudge2* suggest that the decision-maker should increase the reservation wage. Similarly to *Nudge1*, *Nudge1+2* has no significant impact on the search effort or the payoffs.

***Result 4*. *Nudge1 has a positive impact on the reservation wage*, *but it does not significantly affect the search effort and the payoffs of individuals*. *Our results partially confirm Hypothesis 2a***.

In sum, our results suggest that reducing the search costs is an effective way of inducing individuals to search harder for a job and it increases the individual payoffs, while nudging is more effective in increasing the reservation wage. This suggest that the two policy measures should be combined in order to improve the job seekers’ welfare.

### 3.4. Experimental results II: Alleviating the impact of sunk-cost fallacy

In this section, we analyze how our interventions affect the impact of the sunk-cost fallacy on the choice of reservation wage, which shows a decreasing trend in *Baseline*. To this end, we regress the reservation wage on the number of the period when the decision was submitted, controlling for the duration of the search spell and individual characteristics. We run a separate regression for each relevant treatment: *Baseline*, *LowCost*, *Nudge2 and Nudge1+2*. The results are displayed in Table 5. We focus on the coefficient of period in these regressions, which captures how individuals change their reservation wage after an unsuccessful search period.

**Table 5:** The change of reservation wage over the search spell.

	(1)	(2)	(3)	(4)
	Baseline	Low cost	Nudge 2	Nudge 1+2
Period	-0.417***	-0.195	0.095	-0.148
	(0.131)	(0.153)	(0.156)	(0.091)
Duration	0.418***	0.199**	0.154	0.117
	(0.114)	(0.079)	(0.104)	(0.084)
Round	-0.157	-0.938***	0.077	-0.111
	(0.233)	(0.245)	(0.239)	(0.185)
Age	1.191**	0.387	-0.112	0.793*
	(0.490)	(0.538)	(0.563)	(0.461)
Female	-0.525	-3.288	-0.226	0.153
	(2.646)	(2.703)	(2.627)	(2.388)
Cog. Refl. Test	2.887**	2.108**	1.191	1.915
	(1.149)	(1.070)	(1.121)	(1.225)
Risk (investment)	0.037	0.108**	0.158***	0.167***
	(0.046)	(0.049)	(0.042)	(0.044)
Vocational School	11.494***	-5.540	1.976	0.827
	(4.001)	(5.701)	(5.716)	(5.125)
Bachelor’s degree	-2.837	-5.100**	4.555	2.057
	(2.973)	(2.511)	(3.026)	(2.805)
Master’s degree	-1.800	2.991	4.525	-4.891
	(4.168)	(4.292)	(4.745)	(4.013)
Part-time work	-5.731*	3.175	2.259	4.362
	(3.348)	(2.899)	(3.417)	(3.216)
Full-time work	-6.516	1.428	-3.633	6.215*
	(4.061)	(4.702)	(3.571)	(3.218)
Work experience (years)	-0.021	-0.234	1.000**	-0.430
	(0.574)	(0.471)	(0.479)	(0.560)
Num. previous experiments	0.330***	0.188	-0.252*	-0.021
	(0.099)	(0.151)	(0.132)	(0.145)
Student	5.758*	0.551	1.248	9.449***
	(3.280)	(2.737)	(3.071)	(2.811)
Constant	8.732	32.994**	33.579**	12.766
	(11.971)	(13.387)	(13.346)	(11.872)
Chi2	52.514	56.540	40.947	58.871
p-value	0.000	0.000	0.000	0.000
R2 within	0.010	0.015	0.008	0.001
R2 between	0.169	0.175	0.153	0.219
R2 overall	0.065	0.066	0.117	0.114
Number of observations	5,429	4,238	4,647	6,260

*Note*: Random-effects regression model with standard errors clustered at the individual level (shown in parenthesis). Sample: *Baseline* (column 1), *LowCost* (column 2), *Nudge 2* (column 3), *Nudge1+2* (column 4). Omitted categories: Male, High school graduate, Not working, Not a student. *** denotes significance at the 1% level,

** at the 5% level,

* at the 10% level.

As discussed above, the coefficient of period is negative and statistically significant in *Baseline* (see column 1 of Table 5). Hypothesis 3b stated that decreasing the search costs as in *LowCost* may eliminate this trend as individuals will accumulate lower search costs due to sunk-cost fallacy. We find evidence for this effect as the coefficient of period is not statistically significant in the regression using the sample from *LowCost* (see column 2 of Table 5). This finding confirms the predictions of Hypothesis 3b.

Next, we turn to *Nudge2*, which warns individuals about the negative payoff effects of reducing the reservation wage after an unsuccessful search period. We find that in the regression using the sample from *Nudge2*, the coefficient of period is positive but not statistically significant (see column 3 in Table 5). This suggests that nudging was indeed effective in lowering the impact of the sunk-cost fallacy and confirms Hypothesis 2b. Finally, we do the same analysis for *Nudge1+2*, and again find a statistically insignificant coefficient of period in the regression (see column 4 in Table 5). This shows that nudging works even if it is combined with other messages. We note that we have run a similar regression for the Nudge1 treatment as a placebo test, and we find a significant and negative coefficient of period for this treatment, similarly to Baseline. The regression is available in the S1 Appendix (see Table A5 in the S1 Appendix).

***Result 5*. *Lowering the search costs and nudging are both effective in reducing the impact of the sunk-cost fallacy on the choice of reservation wages*. *Our results confirm Hypothesis 2b and 3b***.

## 4. Conclusion

In this paper, we study the effectiveness of policy interventions in alleviating the impact of behavioral biases on job search. We present the results of a stylized search experiment with an infinite horizon whereby individuals choose costly search effort and reservation wage. We show that job seekers choose a lower search effort and a lower reservation wage than the optimal values of a risk-averse decision-maker who exponentially discounts future payoffs. We show that this deviation can be partially explained by quasi-hyperbolic discounting and present bias. In addition, individuals increase the search effort level and decrease the reservation wage over the search spell, whereas the optimal choice in an infinite-horizon framework is to keep those values constant. These deviations from the optimal choices are consistent with a model where individuals exhibit the sunk-cost fallacy. We show that both quasi-hyperbolic discounting and the sunk-cost fallacy lead to welfare loss for the individual.

We study the impact of two policy interventions, the reduction of search costs and nudging, with the aim to increase individual welfare. Our nudging messages inform individuals about the nature of behavioral biases and suggest a course of action to improve their payoffs. We apply two types of messages, one targeting the consequences of present bias, the other the impact of the sunk-cost fallacy. We obtain that reducing the search costs effectively increases the investment in search effort, leading to higher individual welfare, but has no significant impact on the reservation wage. As for the behavioral intervention, our nudging message targeting present bias can significantly increase the reservation wage. However, it has no significant impact on the search effort or individual welfare. In addition, we find that both the search cost reduction and nudging can correct the declining tendency of the reservation wage during the search spell.

Our results suggest that adding behavioral interventions to active labor market policies is an effective and inexpensive way of improving the job search outcomes of unemployed workers. In some cases, however, the impact of these measures is not strong enough to positively affect all outcome variables of interest, in particular, individual welfare. Therefore, we also point out that these interventions need to be combined with more traditional policy measures that lead to the reduction of search costs, including job search assistance and information provision.

## Supporting information

S1 Appendix (DOCX)

S1 Data (ZIP)

S1 File Experimental instructions—baseline. (DOCX)
