# Supplementary material for: Alleviating behavioral biases at job search: Do nudges work?
Source: PLoS One. 2022 Apr 6;17(4):e0266105. doi: 10.1371/journal.pone.0266105 (PMC8985956; doi:10.1371/journal.pone.0266105)
Supplement: S1 Appendix — (DOCX) [file pone.0266105.s001.docx]

**Supporting Information to**

**Alleviating behavioral biases at job search: Do nudges work?**

Gergely Horvath^^[[1]](#footnote-1)^^

*Division of Social Sciences, Duke Kunshan University*

# Experimental procedures and sample

The experiment was conducted online through the Prolific website ([1]) between May and September 2020. There is broad evidence that online experiments are reliable and produce comparable results to lab experiments ([2], [3], [4], [5], [6], [7]). They allow researchers to collect data with a larger sample size in a timely and cost-effective manner. Moreover, this experiment consists of an individual decision-making task, therefore it is not affected by some of the difficulties that are present in interactive online experiments (see [8] for details). The experiment was programmed in o-Tree ([9]).

We recruited subjects through Prolific with the only restriction being that subjects should have at least a high school diploma. Once participants started the study on Prolific, they were presented with the instructions (these are available attached to this article). After reading the instructions, participants answered a set of control questions. They were not allowed to proceed further until all questions had been answered correctly. Then, they participated in the eight rounds of the job search task, after which they filled out a questionnaire about demographic information, risk and time preferences, and a cognitive reflection test. After that, participants exited the study and received their payment online. The average payment was £4.77 including a £2.5 show-up fee. We note that the payoffs are somewhat lower compared to lab experiments, which is a characteristic of online experiments ([8]). On the one hand, the experiment was shorter (35 minutes on average) than a typical lab experiment, which justifies the lower payments. On the other hand, participants made suboptimal decisions, as described in the main text, which decreased their payoffs compared to the potential payoffs they could have earned if they followed the optimal search strategy. We also note that online experiments typically have larger show-up fees as percentage of the total payoffs. This is to attract participants within the recruitment platform to the experiment ([8]).

There were in total 740 participants in the study, the breakdown by treatments is as follows: 157 in *Baseline,* 145 in *LowCost*, 141 in *Nudge1,* 144 in *Nudge2*, and 153 in *Nudge1+2*. The participants submitted 25,373 search strategies in total over the eight rounds of the experiment.

Table A1 summarizes the characteristics of the experimental sample. In terms of demographics, the average age of the participants was 24.2 years old, 42.3% of the participants were female. Around 56.2% of the participants had completed at least a bachelor’s degree, while 58.8% were students. Table A1 reports the participants’ country of origin, focusing on countries that are more represented.

In the post-experimental questionnaire, we elicited time and risk preferences that may have influenced the behavior of the participants in the experiment. Regarding risk aversion, we used two different methods. Firstly, we asked participants to evaluate themselves by answering the question *“Are you generally a person who is fully prepared to take risks or do you try to avoid taking risks? Rate yourself from 0 to 10, where 0 means 'unwilling to take any risk' and 10 means 'fully prepared to take risks’.*” This question is easy to understand and captures general risk-taking ([10]). The average score of all participants was 5.7. Secondly, we asked participants to make an incentivized investment decision by presenting them with the following scenario originating from [11]: *“Imagine you have 100 points. You can invest some of it in a risky investment. If you invest Y in the risky investment, the investment return will be either 2.5*Y or 0 with equal probability. You may think of this as if the investment return was decided by a coin toss. The amount not invested in the risky investment is 100-Y. Please, enter below how much you would like to invest in the risky investment. After this, the computer will toss a coin and determine the investment return. Your payoff will be 100-Y+the investment return in points. This will be added to the payoffs you earned in Part 1 of the Experiment. The amount you would like to invest in the risky investment is:”* Note that risk-neutral individuals should invest all their endowment in the risky asset, while risk-averse individuals should invest less, depending on their degree of risk aversion. In this task, the average amount invested in the risky asset was 57.9. This is the optimal choice of an individual with CRRA utility function when $\gamma=0.27$. Hence, we used this value when computing the theoretical predictions (see in the main text).

We elicited time preferences by asking participants to choose between a payment today and a payment in five weeks, using a simple version of the double multiple-price list method ([12]). Participants completed six choices where the future payment was always £16 while today’s payment was decreasing over the six choices from £15.3 to £8.8 ([13]). Today’s payment thus became less and less attractive as the participant advanced from choice 1 to choice 6. We measure time preference through the switching point: the number of the choice task in which the participant chooses the future payment instead of today’s payment for the first time. This task was not incentivized. The overwhelming majority of subjects (93.1%) answered the six questions rationally and consistently, that is, switching only once. The average switching point was 3.8. The remaining subjects showed inconsistent behavior. We do not exclude these individuals from our sample, instead, we control for this behavior in the regression analyses in the Appendix by introducing a dummy variable for this behavior.

We also add an incentivized cognitive reflection task to the post-experimental survey. We use the standard three questions first proposed by [14]. Each correct answer was awarded 50 points. The average number of correct answers was 1.7.

#

**Table A1: Sample characteristics**

| **Characteristic** | **Mean**  **(Std. dev)** | **Characteristic** | **Mean**  **(Std. dev)** |
| --- | --- | --- | --- |
| Age | 24.168 | Work experience | 2.674 |
|  | (3.305) |  | (2.994 |
| Female | 0.423 | Exp. Experience | 5.242 |
|  | (0.494) |  | (8.325 |
| Cog. Reflection | 1.716 | *Country of origin* |  |
|  | (1.127) | UK | 0.028 |
| Risk (self-evaluation) | 5.726 |  | (0.166) |
|  | (1.989) | Poland | 0.189 |
| Risk2 (investment) | 57.930 |  | (0.392) |
|  | (27.468) | USA | 0.064 |
| Time pref., switching point | 3.758 |  | (0.244) |
|  | (1.819) | Portugal | 0.146 |
| *Education & work experience* | |  | (0.353) |
| High school | 0.380 | Spain | 0.039 |
|  | (0.486) |  | (0.194) |
| Vocational school | 0.049 | Greece | 0.059 |
|  | (0.215) |  | (0.237) |
| Bachelor’s degree | 0.393 | Hungary | 0.034 |
|  | (0.489) |  | (0.181) |
| Master’s degree | 0.166 | Canada | 0.009 |
|  | (0.373) |  | (0.097) |
| Student | 0.588 | Italy | 0.119 |
|  | (0.493) |  | (0.324) |
| Part-time work | 0.226 | Mexico | 0.069 |
|  | (0.418) |  | (0.253) |
| Full-time work | 0.272 | Other countries | 0.243 |
|  | (0.445) |  | (0.429) |

# Welfare effects in the behavioral models of job search

We illustrate the welfare effects of quasi-hyperbolic discounting and the sunk-cost fallacy by solving the models in Section 2.2.1 and 2.2.2 for the parameter values of the experiment.

Firstly, we show that in the quasi-hyperbolic discounting model increasing the search intensity and reservation wage will increase the discounted expected utility and payoffs. This is shown in the left-hand panels of Figure 1, which depict welfare and payoffs as functions of increasing the search intensity and reservation wage simultaneously starting from the values chosen by the individual according to the quasi-hyperbolic discounting model ($R^{*}=55$, $s^{*}=54$). We can observe that increasing search intensity and reservation wage raises individual welfare at the beginning before welfare peaks and starts to decline. This illustrates that policy interventions that increase search intensity and reservation wage can improve individual welfare and payoffs.

Secondly, we illustrate the effects of the sunk-cost fallacy in the right-hand panels of Figure 1, which show the evolution of welfare and payoffs over the search spell for different degrees of the sunk-cost fallacy captured by the value of $\alpha$ in the model presented in Section 2.2.2. We can observe that the sunk-cost fallacy reduces welfare and payoffs. Note that this effect is independent of the way of discounting.

**Figure A1: Welfare and payoffs in the quasi-hyperbolic and the sunk-cost fallacy models**

**
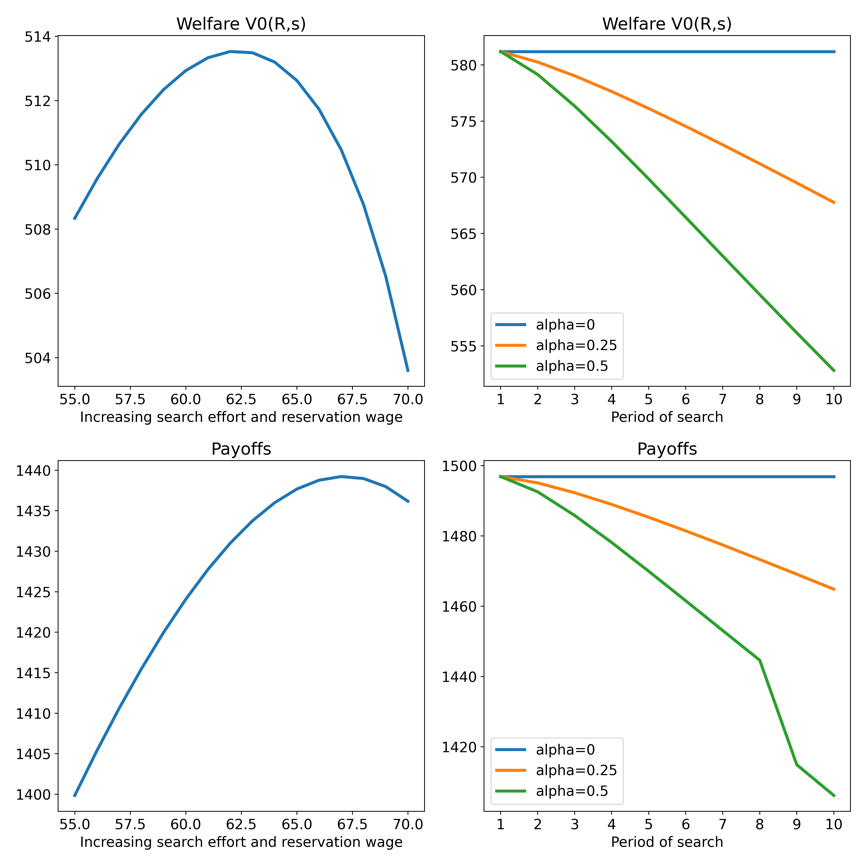
**

*Note:* We depict expected discounted utility (equation 2) and total payoffs from a round (equation 6) for solving the models in Sections 2.2.1 and 2.2.2 for the parameter values applied in the experiment. For the quasi-hyperbolic discounting model, we show how welfare and payoffs change when the levels of search intensity and reservation wages are simultaneously increased from the optimal values chosen by the individual. This is depicted on the two left-hand side panels. For the sunk-cost fallacy model, we show the evolution of welfare and payoffs over the search spell. This is depicted on the two right-hand side panels.

# Additional Figures

**Figure A2: The impact of the sunk-cost fallacy on the choice of reservation wages and search intensity**


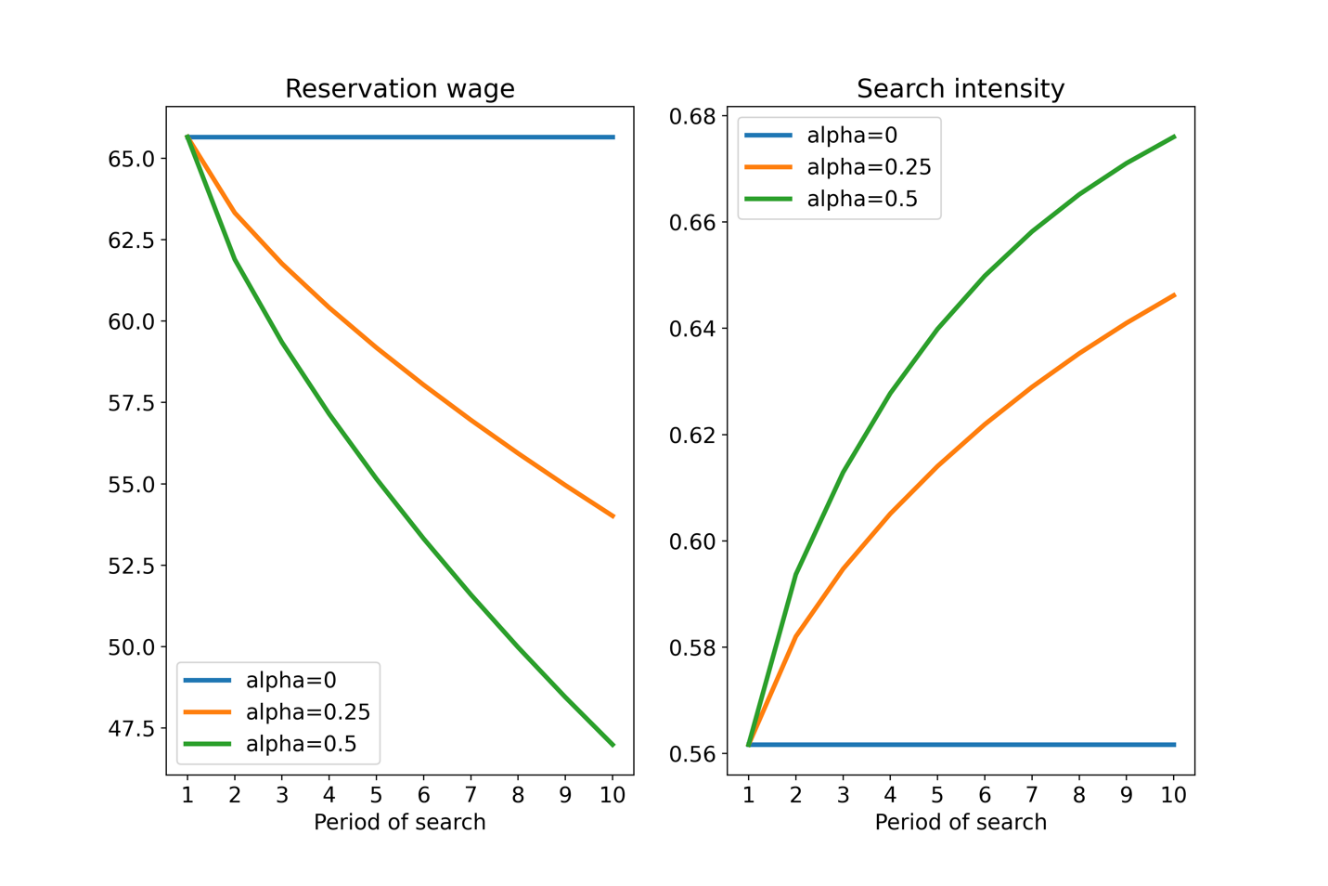


*Note:* We solve the model in Section 2.2.2. for the parameter values of the experiment and different values of the strength of the sunk-cost fallacy $\alpha.$

**Figure A3: Distribution of key outcomes in *Baseline***


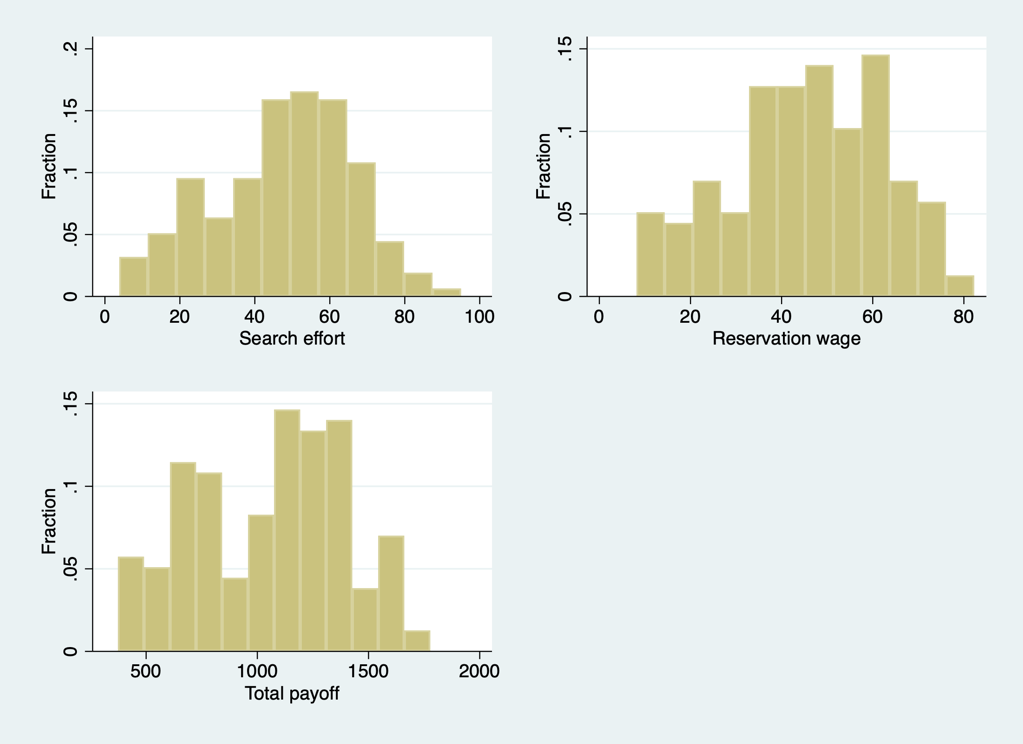


*Note:* The figure shows the distribution of search effort, reservation wage and total payoffs in a round for *Baseline*. It shows the distribution of individual averages of these outcomes.

**Figure A4: Evolution of search effort over the search spell for a given search duration**

**
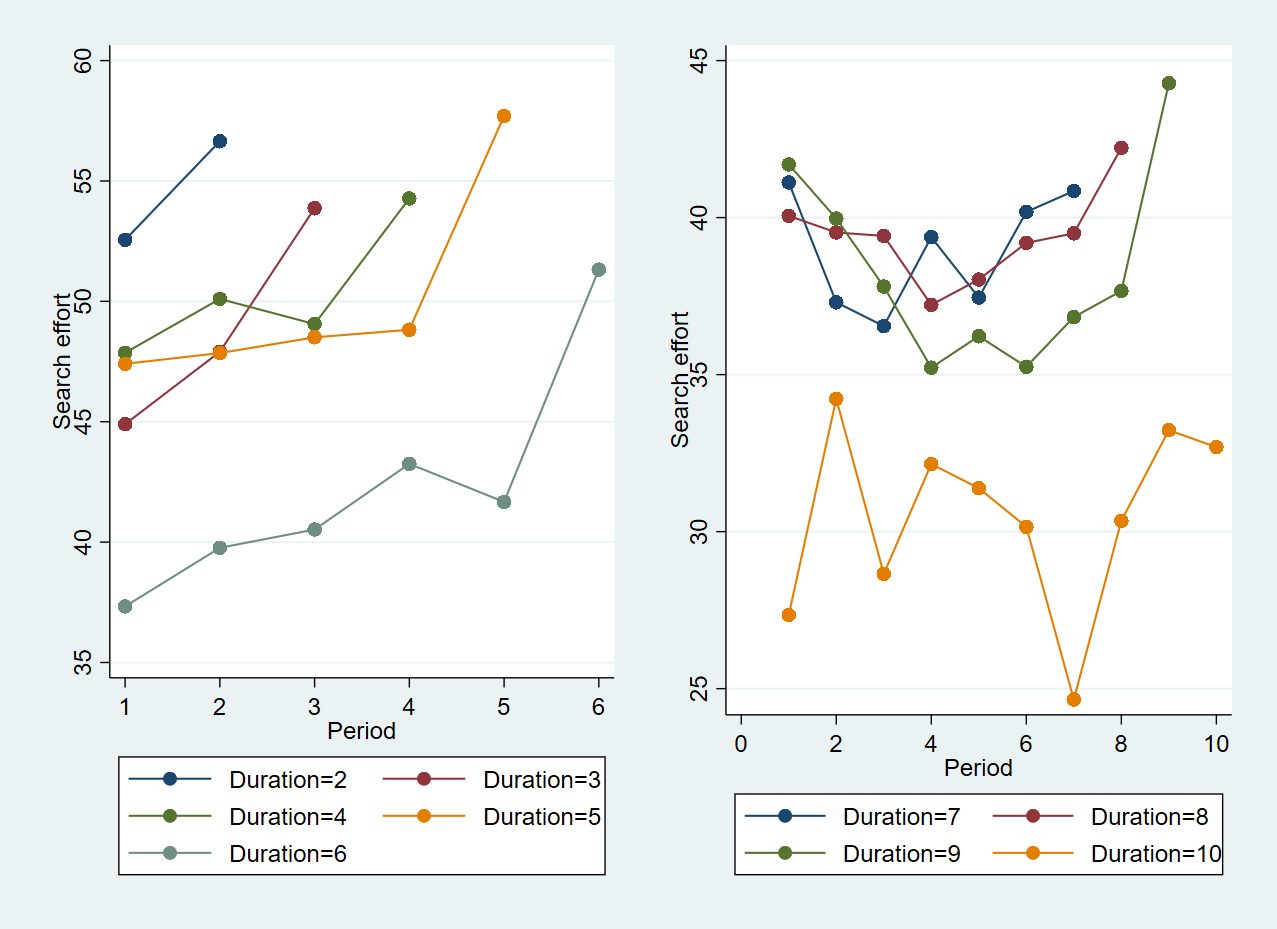
**

*Note:* The figure shows the average value of search effort as function of the search period for different values of search duration. We note that 80% of the search spells are represented by the left panel as they ended within 6 periods.

**Figure A5: Evolution of reservation wage over the search spell for a given search duration**

**
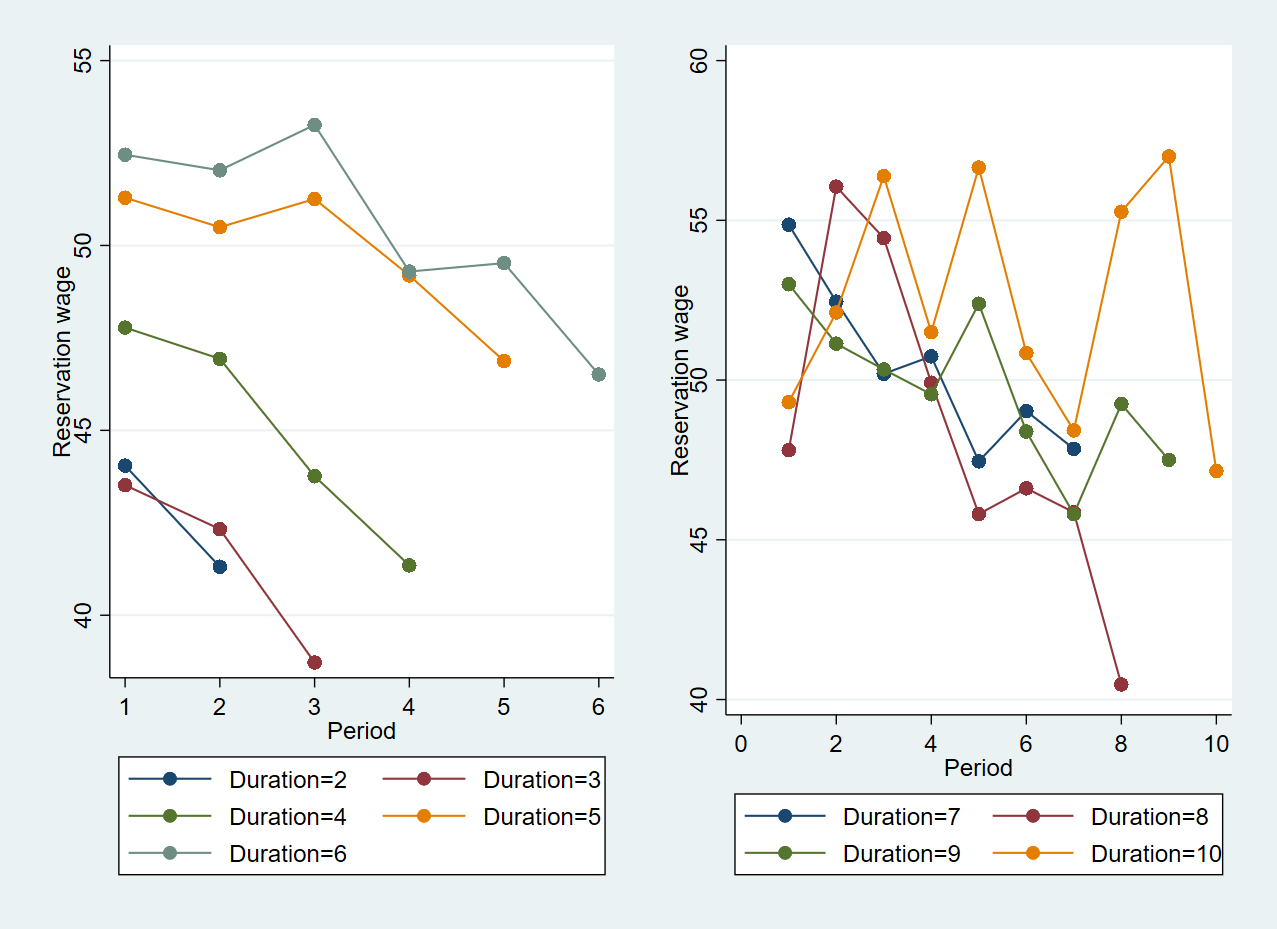
**

*Note:* The figure shows the average value of reservation wage as function of the search period for different values of search duration. We note that 80% of the search spells are represented by the left panel as they ended within 6 periods.

**Figure A6: Evolution of reservation wage and search intensity over rounds of job search task in *Baseline***

**
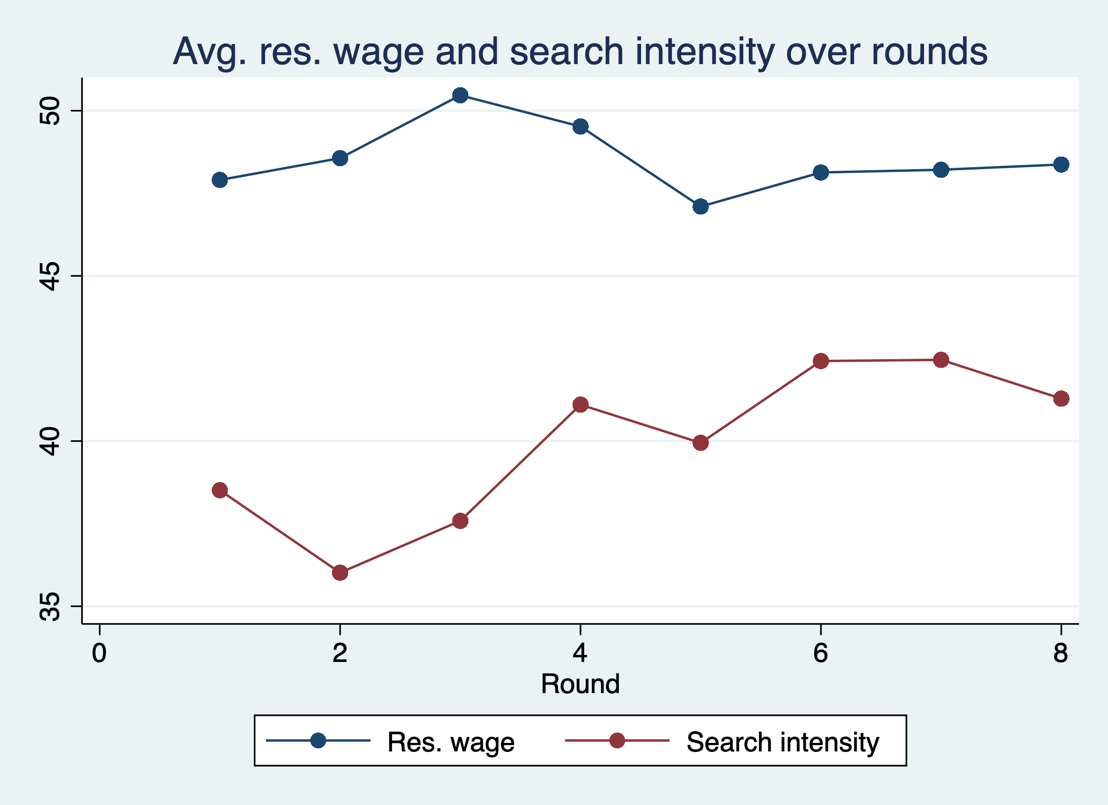
**

*Note:* The figure depicts the average reservation wage and the average search intensity for a given round of job search task.

#

# Additional Tables and Regression Analyses

We provide additional results, including non-parametric tests for between-treatment comparisons between *Baseline* and the other treatments. We also provide further regression analyses that replicate the findings of the main text with a broader set of controls, in particular, controlling for our measure of time preference.

**Table A2: Theoretical predictions vs. experimental data and treatment comparisons**

|  | ***LowCost*** | | | | ***Comparison to Baseline*** | |
| --- | --- | --- | --- | --- | --- | --- |
|  | **Prediction** | **Mean** | **Std. dev.** | **# of obs.** | **z-score** | **p-value** |
| Search effort | 74 | 54.71 | 19.72 | 145 | -3.75 | 0.000 |
| Res. Wage | 69 | 47.2 | 14.87 | 145 | -0.73 | 0.464 |
| Total payoff | 1500.15 | 1019.8 | 10.22 | 145 | 0.95 | 0.344 |
|  | ***Nudge1*** | | | | ***Comparison to Baseline*** | |
|  | **Prediction** | **Mean** | **Std. dev.** | **# of obs.** | **z-score** | **p-value** |
| Search effort | 57 | 52.33 | 18.21 | 141 | -2.33 | 0.020 |
| Res. Wage | 65 | 50.55 | 16.76 | 141 | -2.22 | 0.027 |
| Total payoff | 1437.95 | 925.2 | 10.99 | 141 | 3.89 | 0.000 |
|  | ***Nudge2*** | | | | ***Comparison to Baseline*** | |
|  | **Prediction** | **Mean** | **Std. dev.** | **# of obs.** | **z-score** | **p-value** |
| Search effort | 57 | 45.15 | 18.32 | 144 | 0.94 | 0.349 |
| Res. Wage | 65 | 47.75 | 16.09 | 144 | -0.99 | 0.321 |
| Total payoff | 1437.95 | 959.4 | 9.28 | 144 | 2.60 | 0.009 |
|  | ***Nudge1+2*** | | | | ***Comparison to Baseline*** | |
|  | **Prediction** | **Mean** | **Std. dev.** | **# of obs.** | **z-score** | **p-value** |
| Search effort | 57 | 49.04 | 18.97 | 153 | -0.80 | 0.425 |
| Res. Wage | 65 | 51.35 | 16.46 | 153 | -2.96 | 0.003 |
| Total payoff | 1437.95 | 996.9 | 9.2 | 153 | 2.29 | 0.022 |

*Note:* Non-parametric tests to compare the experimental data from the different treatments to *Baseline*. We form independent observations by averaging the outcome variables for each individual over all search periods and rounds.

**Table A3: Changes in search behavior over the search spell in *Baseline***

|  | (1) | (2) | (3) | (4) |
| --- | --- | --- | --- | --- |
|  | Reservation wage | Reservation wage | Search effort | Search effort |
| Period | -0.417*** | -0.429*** | 0.217* | 0.320** |
|  | (0.131) | (0.156) | (0.128) | (0.128) |
| Duration | 0.417*** | 0.473*** | -0.476*** | -0.668*** |
|  | (0.114) | (0.134) | (0.181) | (0.150) |
| Round | -0.158 | -0.161 | 0.493** | 0.564** |
|  | (0.233) | (0.250) | (0.225) | (0.241) |
| Age | 1.114** | 1.162** | 0.065 | -0.074 |
|  | (0.527) | (0.509) | (0.578) | (0.507) |
| Female | -0.494 | -0.781 | -5.062* | -3.487 |
|  | (2.683) | (2.974) | (2.683) | (2.694) |
| Cog. Refl. Test | 3.468*** | 3.067** | 4.880*** | 5.738*** |
|  | (1.224) | (1.366) | (1.234) | (1.221) |
| Risk pref. (self-evaluation) | 0.738 | 0.752 | -0.560 | -0.178 |
|  | (0.918) | (0.935) | (0.915) | (0.890) |
| Risk (investment) | 0.044 | 0.019 | 0.055 | 0.038 |
|  | (0.046) | (0.049) | (0.048) | (0.047) |
| Vocational School | 9.401** | 11.819** | -4.406 | -11.304* |
|  | (4.445) | (4.829) | (6.545) | (6.770) |
| Bachelor’s degree | -1.950 | -2.719 | 2.046 | 1.198 |
|  | (3.024) | (3.212) | (2.926) | (2.781) |
| Master’s degree | -1.347 | -1.948 | 0.799 | 0.733 |
|  | (4.208) | (4.125) | (4.296) | (4.025) |
| Part-time work | -7.628** | -4.458 | 1.631 | 0.423 |
|  | (3.312) | (3.589) | (3.493) | (3.629) |
| Full-time work | -8.336** | -5.834 | 0.487 | -1.512 |
|  | (4.020) | (4.274) | (3.826) | (3.868) |
| Work experience (years) | 0.170 | -0.041 | -0.937 | -0.704 |
|  | (0.627) | (0.617) | (0.629) | (0.601) |
| Num. previous experiments | 0.277** | 0.333*** | 0.041 | 0.021 |
|  | (0.121) | (0.107) | (0.120) | (0.115) |
| Student | 6.220* | 6.604* | -3.614 | -4.264 |
|  | (3.372) | (3.626) | (3.170) | (3.238) |
| Time preference |  | -0.168 |  | 0.099 |
|  |  | (0.817) |  | (0.780) |
| Constant | 7.444 | 5.821 | 46.301*** | 46.083*** |
|  | (14.760) | (13.764) | (15.808) | (13.957) |
| Time preference dummies ddummies | Yes | No | Yes | No |
| Chi2 | 71.189 | 50.870 | 84.421 | 123.821 |
| p-value | 0.000 | 0.000 | 0.000 | 0.000 |
| R2 within | 0.010 | 0.011 | 0.017 | 0.026 |
| R2 between | 0.217 | 0.176 | 0.312 | 0.338 |
| R2 overall | 0.083 | 0.070 | 0.227 | 0.248 |
| Number of observations | 5,429 | 4,954 | 5,429 | 4,954 |

*Note:* Random-effects regression model with standard errors clustered at the individual level (shown in parenthesis). Sample: *Baseline* treatment. Omitted categories: Male, High school graduate, Not working, Not a student. *** denotes significance at the 1% level, ** at the 5% level, * at the 10% level. In column 1 and 3, the sample includes all observations and time preferences are represented by a set of dummies that capture switching points, including a separate dummy for multiple switching points. In column 2 and 4, the sample includes observations with a unique switching point in the time preference elicitation task and time preference is represented by the switching point.

We briefly comment on the impact of some individual characteristics on the choice of reservation wage and search effort based on the regression coefficients from Table A4 in the Appendix. We do not observe significant gender differences in the choice of search effort or reservation wage, albeit female set lower values for these variables, the difference is not statistically significant. Higher score in the cognitive reflection test is associated with a higher search effort and a higher reservation wage, suggesting that participants who reflect more before making a decision do better at this task. One interesting question is whether individuals learn to play better strategies over repetitions of the decision problem. The coefficient of search round is positive and statistically significant in the regression of search effort (see column 3 and 4), suggesting that individuals learn to set a higher search effort over time. In contrast, we do not observe such learning for reservation wage, as the coefficient of round in that regression is not statistically significant (see column 1 and 2). The elicited measures of risk aversion have no significant impact on the choices of the participants. We do not find a significant relationship between time preference and the choice of reservation wage or search effort either (see column 2 and 4). This may be because the elicitation of time preference was not incentivized. We also note that the simple measure of time preference elicited here does not capture present bias because it only requires individuals to choose between a smaller present and a larger future gain, representing general impatience.

**Table A4: Treatment effects**

|  | (1) | (2) | (5) |
| --- | --- | --- | --- |
|  | **Search effort** | **Res. Wage** | **Total payoff** |
| *LowCost* | 6.138*** | 1.373 | 56.638** |
|  | (2.072) | (1.795) | (23.973) |
| *Nudge1* | 3.095 | 4.093** | 23.604 |
|  | (2.042) | (1.935) | (23.553) |
| *Nudge2* | -3.947** | 1.342 | 35.713 |
|  | (1.949) | (1.834) | (25.813) |
| *Nudge1+2* | 1.438 | 6.006*** | 13.462 |
|  | (1.911) | (1.779) | (21.660) |
| Period | -0.067* | 0.004 | 66.441*** |
|  | (0.035) | (0.058) | (1.035) |
| Round | 0.809*** | -0.185* | -4.164** |
|  | (0.109) | (0.105) | (2.075) |
| Age | -0.079 | 0.706*** | 4.416 |
|  | (0.319) | (0.239) | (3.604) |
| Female | -3.052** | -0.819 | -15.033 |
|  | (1.318) | (1.234) | (15.757) |
| Cog. Refl. Test | 3.943*** | 1.725*** | 16.458** |
|  | (0.598) | (0.530) | (7.196) |
| Risk pref. (self-evaluation) | -0.770** | 0.254 | -2.754 |
|  | (0.372) | (0.318) | (3.916) |
| Risk (investment) | 0.055** | 0.106*** | 0.417 |
|  | (0.025) | (0.022) | (0.298) |
| Vocational School | -0.202 | 1.826 | 18.128 |
|  | (3.081) | (2.515) | (37.168) |
| Bachelor’s degree | 2.416 | -1.109 | -0.348 |
|  | (1.563) | (1.360) | (17.685) |
| Master’s degree | 1.397 | -0.035 | 4.559 |
|  | (2.154) | (1.999) | (25.923) |
| Part-time work | 0.415 | 1.305 | -2.314 |
|  | (1.718) | (1.498) | (18.992) |
| Full-time work | -1.888 | -0.422 | -20.145 |
|  | (1.990) | (1.775) | (22.678) |
| Work experience (years) | -0.353 | -0.011 | -6.027* |
|  | (0.324) | (0.247) | (3.552) |
| Num. previous experiments | -0.009 | 0.038 | -0.648 |
|  | (0.083) | (0.073) | (0.843) |
| Student | 3.577** | 3.612*** | -10.098 |
|  | (1.609) | (1.384) | (17.542) |
| Constant | 36.382*** | 12.724* | -330.648*** |
|  | (8.161) | (6.690) | (92.758) |
| Time preference dummies | Yes | Yes | Yes |
| Chi2 | 261.157 | 119.671 | 5,792.195 |
| p-value | 0.000 | 0.000 | 0.000 |
| R2 within | 0.012 | 0.001 | 0.843 |
| R2 between | 0.196 | 0.112 | 0.598 |
| R2 overall | 0.136 | 0.062 | 0.825 |
| Number of observations | 25,350 | 25,350 | 5,798 |

*Note:* Random-effects regression model with standard errors clustered at the individual level (shown in parenthesis). Sample: all treatments. Omitted categories: *Baseline* treatment, Male, High-school graduate, Not working, not a student. *** denotes significance at the 1% level, ** at the 5% level, * at the 10% level. Time preferences are represented by a set of dummy variables, with a separate dummy variable for each switching point in the elicitation task. We also include a dummy variable for inconsistent choice (that is, multiple switching point).

**Table A5: The change of reservation wage over the search spell**

|  | **(1)** | **(2)** | **(3)** | **(4)** | **(5)** |
| --- | --- | --- | --- | --- | --- |
|  | **Baseline** | **Low cost** | **Nudge 2** | **Nudge 1+2** | **Nudge 1** |
| Period | -0.417*** | -0.195 | 0.095 | -0.148 | -0.472*** |
|  | (0.131) | (0.153) | (0.156) | (0.092) | (0.144) |
| Duration | 0.417*** | 0.199** | 0.154 | 0.121 | 0.402*** |
|  | (0.114) | (0.079) | (0.105) | (0.084) | (0.128) |
| Round | -0.158 | -0.940*** | 0.072 | -0.108 | 0.206 |
|  | (0.233) | (0.245) | (0.239) | (0.185) | (0.255) |
| Age | 1.114** | 0.357 | 0.372 | 0.928* | 0.739 |
|  | (0.527) | (0.511) | (0.606) | (0.486) | (0.561) |
| Female | -0.494 | -3.470 | -0.790 | -0.960 | -1.307 |
|  | (2.683) | (2.759) | (2.591) | (2.430) | (3.034) |
| Cog. Refl. Test | 3.468*** | 2.147** | 1.340 | 1.202 | 0.264 |
|  | (1.224) | (1.094) | (1.231) | (1.174) | (1.254) |
| Risk pref. (self-evaluation) | 0.738 | -0.031 | 1.523** | -0.050 | -1.111 |
|  | (0.918) | (0.596) | (0.699) | (0.620) | (0.732) |
| Risk (investment) | 0.044 | 0.118** | 0.145*** | 0.169*** | 0.109** |
|  | (0.046) | (0.051) | (0.043) | (0.044) | (0.053) |
| Vocational School | 9.401** | -5.136 | 2.787 | -2.664 | 7.048 |
|  | (4.445) | (5.808) | (5.958) | (4.948) | (5.403) |
| Bachelor’s degree | -1.950 | -5.568** | 3.745 | -1.014 | -5.973* |
|  | (3.024) | (2.665) | (3.033) | (2.819) | (3.201) |
| Master’s degree | -1.347 | 2.382 | 2.762 | -8.113** | 2.454 |
|  | (4.208) | (4.690) | (4.583) | (3.873) | (4.193) |
| Part-time work | -7.628** | 3.470 | 2.750 | 4.894 | 2.428 |
|  | (3.312) | (2.997) | (3.343) | (3.060) | (3.778) |
| Full-time work | -8.336** | 1.297 | -4.021 | 5.221* | 3.506 |
|  | (4.020) | (5.088) | (3.443) | (3.036) | (4.353) |
| Work experience (years) | 0.170 | -0.198 | 0.576 | -0.056 | -0.593 |
|  | (0.627) | (0.498) | (0.489) | (0.599) | (0.584) |
| Num. previous experiments | 0.277** | 0.199 | -0.305*** | -0.068 | 0.089 |
|  | (0.121) | (0.163) | (0.118) | (0.146) | (0.189) |
| Student | 6.220* | 0.641 | 2.068 | 10.391*** | 0.721 |
|  | (3.372) | (2.833) | (3.100) | (2.610) | (3.463) |
| Constant | 7.444 | 30.837** | 11.678 | 2.399 | 29.476* |
|  | (14.760) | (13.124) | (18.049) | (12.387) | (15.621) |
| Time preference dummies | Yes | Yes | Yes | Yes | Yes |
| Chi2 | 71.189 | 64.512 | 55.737 | 102.289 | 62.604 |
| p-value | 0.000 | 0.000 | 0.000 | 0.000 | 0.000 |
| R2 within | 0.010 | 0.015 | 0.008 | 0.001 | 0.010 |
| R2 between | 0.217 | 0.188 | 0.203 | 0.308 | 0.182 |
| R2 overall | 0.083 | 0.067 | 0.132 | 0.141 | 0.109 |
| Number of observations | 5,429 | 4,238 | 4,647 | 6,260 | 4,776 |

*Note:* Random-effects regression model with standard errors clustered at the individual level (shown in parenthesis). Sample: *Baseline* (column 1), *LowCost* (column 2), *Nudge 2* (column 3), *Nudge1+2* (column 4). Omitted categories: Male, High school graduate, Not working, Not a student. *** denotes significance at the 1% level, ** at the 5% level, * at the 10% level. Time preferences are represented by a set of dummy variables, with a separate dummy variable for each switching point in the elicitation task. We also include a dummy variable for inconsistent choice (that is, multiple switching point).

# References

1. Palan S, Schitter C. Prolific. ac—A subject pool for online experiments. Journal of Behavioral and Experimental Finance. 2018 Mar 1;17:22-7.
2. Amir O, Rand DG, Gal YA. Economic games on the internet: The effect of $1 stakes. PloS one. 2012 Feb 21;7(2):e31461.
3. Horton JJ, Rand DG, Zeckhauser RJ. The online laboratory: Conducting experiments in a real labor market. Experimental economics. 2011 Sep;14(3):399-425.
4. Kroher M, Wolbring T. Social control, social learning, and cheating: Evidence from lab and online experiments on dishonesty. Social Science Research. 2015 Sep 1;53:311-24.
5. Paolacci G, Chandler J, Ipeirotis PG. Running experiments on amazon mechanical turk. Judgment and Decision making. 2010 Jun 24;5(5):411-9.
6. Rand DG. The promise of Mechanical Turk: How online labor markets can help theorists run behavioral experiments. Journal of theoretical biology. 2012 Apr 21;299:172-9.
7. Suri S, Watts DJ. Cooperation and contagion in web-based, networked public goods experiments. PloS one. 2011 Mar 11;6(3):e16836.
8. Arechar AA, Gächter S, Molleman L. Conducting interactive experiments online. Experimental economics. 2018 Mar;21(1):99-131.
9. Chen DL, Schonger M, Wickens C. oTree—An open-source platform for laboratory, online, and field experiments. Journal of Behavioral and Experimental Finance. 2016 Mar 1;9:88-97.
10. Charness G, Gneezy U, Imas A. Experimental methods: Eliciting risk preferences. Journal of Economic Behavior & Organization. 2013 Mar 1;87:43-51.
11. Gneezy U, Potters J. An experiment on risk taking and evaluation periods. The quarterly journal of economics. 1997 May 1;112(2):631-45.
12. Andreoni J, Kuhn MA, Sprenger C. Measuring time preferences: A comparison of experimental methods. Journal of Economic Behavior & Organization. 2015 Aug 1;116:451-64.
13. The six values of future payments were: £15.3, £14.5, £13.7, £12.8, £11.2, £8.8.
14. Frederick S. Cognitive reflection and decision making. Journal of Economic perspectives. 2005 Dec;19(4):25-42.

1. Division of Social Sciences, Duke Kunshan University. Address: Division of Social Sciences, Duke Kunshan University, No. 8 Duke Avenue, Kunshan, Jiangsu Province, China 215316. E-mail address: horvathgergely@gmail.com. Tel: +86 155-01683821. [↑](#footnote-ref-1)
