# Supplementary material for: Alleviating behavioral biases at job search: Do nudges work?
Source: PLoS One. 2022 Apr 6;17(4):e0266105. doi: 10.1371/journal.pone.0266105 (PMC8985956; doi:10.1371/journal.pone.0266105)
Supplement: S1 File — (DOCX) [file pone.0266105.s003.docx]

**Experimental Instructions - Baseline**

**Welcome and thank you for participating in this study.**

**Please, provide your Prolific ID:**

For participating in this experiment you will earn a **fixed show-up fee of 2.5 GBP.** You will earn **additional money** depending on your decisions. This additional amount will paid to you as **Bonus payment.**

**Please, read these instructions carefully so that you fully understand the decisions you need to make.**

**Part 1** of the experiment consists of **8 Rounds**. In each Round you can earn Points. At the end of the experiment, **one Round will be randomly selected** and the Points earned in that Round will determine your additional payment.

In **Part 2** of the experiment you will be asked to fill out a questionnaire, some of the questions will allow you to earn additional Points.

At the end of the experiment, **the Points you earned in Part 1 and Part 2 of the experiment will be converted to GBP** using the exchange rate:

**500 Points = 1 GBP**

This amount plus your show-up fee of 2.5 GBP will be paid to you privately.

**Description of a Round**

Each of the 8 Rounds of the experiment has the same structure. A Round consists of multiple Periods. The **number of Periods** within a Round is not fixed, but it is **randomly determined** by the computer using the following rule: after each Period, there is a 95% chance that the Round continues to the next Period and a 5% chance that the Round ends with the current Period. **Your total payoff from a Round will be the sum of Points you earned over all Periods in the Round.**

You start each Round by searching for offers. Offers are randomly drawn from the set of integer numbers between 1 and 100 Points (1,2,3,…100 Points), with every offer being **equally** **likely**. The value of the offer is important because once you accept an offer, you will earn the value of the offer for the remaining Periods in that Round.

In each Period while you are searching for an offer, you will make **the following** **decisions:**

Firstly, you choose your **minimum acceptable offer,** that is, the lowest offer that you are willing to accept. For example, if your minimum acceptable offer is 59 Points, you will *accept any offer above or equal to 59*, and you will *reject any offer below* 59 Points. Similarly, if your minimum acceptable offer is 79 Points, you will accept any offer above or equal to 79, and you will reject any offer below 79 Points.

Secondly, you decide your **search effort:** it determines the chance that you receive an offer, the higher your search effort is, the larger the chance that you receive an offer. You choose an integer number between 1 and 100. For example, if your search effort is 25, there is a 25% chance that you receive an offer in this Period, and a 75% chance that you do not receive an offer. Or if your search effort is 69, there is a 69% chance that you receive an offer in this Period, and a 31% chance that you do not receive an offer.

A higher search effort is more **costly**. The cost corresponding to all possible search effort levels is shown in Table 1. You will receive **an endowment of 30 Points** for each Period when you search. The cost associated to your search effort level will be deducted from your endowment. Your earnings from this Period will be 30 minus the cost of your search effort. Note that earnings can be negative in a particular Period.

**Example 1:**

The participant chooses the search effort to be equal to 37. The cost of this search effort level can be found in the row starting with ’30’ and the column with the header ‘7’ in Table 1. This shows that the cost is equal to 7.
The participant’s payoff from this Period is 30 - 7 = 23 Points.

**Example 2:**

The participant chooses the search effort to be equal to 75. The cost of this search effort level can be found in the row starting with ’70’ and the column with the header ‘5’ in Table 1. This shows that the cost is equal to 37.
The participant’s payoff from this Period is 30 - 37 = -7 Points.

**TABLE 1: COST OF SEARCH EFFORT**

|  | **0** | **1** | **2** | **3** | **4** | **5** | **6** | **7** | **8** | **9** |
| --- | --- | --- | --- | --- | --- | --- | --- | --- | --- | --- |
| **0** | 5 | 5 | 5 | 5 | 5 | 5 | 5 | 5 | 5 | 5 |
| **10** | 5 | 5 | 5 | 5 | 5 | 5 | 5 | 5 | 5 | 5 |
| **20** | 5 | 5 | 5 | 5 | 5 | 5 | 6 | 6 | 6 | 6 |
| **30** | 6 | 6 | 6 | 6 | 6 | 7 | 7 | 7 | 7 | 7 |
| **40** | 8 | 8 | 8 | 8 | 9 | 9 | 10 | 10 | 10 | 11 |
| **50** | 11 | 12 | 12 | 13 | 14 | 14 | 15 | 16 | 16 | 17 |
| **60** | 18 | 19 | 20 | 21 | 22 | 23 | 24 | 25 | 26 | 28 |
| **70** | 29 | 30 | 32 | 33 | 35 | 37 | 38 | 40 | 42 | 44 |
| **80** | 46 | 48 | 50 | 53 | 55 | 57 | 60 | 62 | 65 | 68 |
| **90** | 71 | 74 | 77 | 80 | 83 | 87 | 90 | 94 | 97 | 101 |

Figure 1 shows a screenshot of the decision screen. You will have 50 seconds to submit your decisions. If you finish earlier, you can click ‘Next’. If you do not submit your decisions within 50 seconds, you have no chance to receive an offer in this Period.


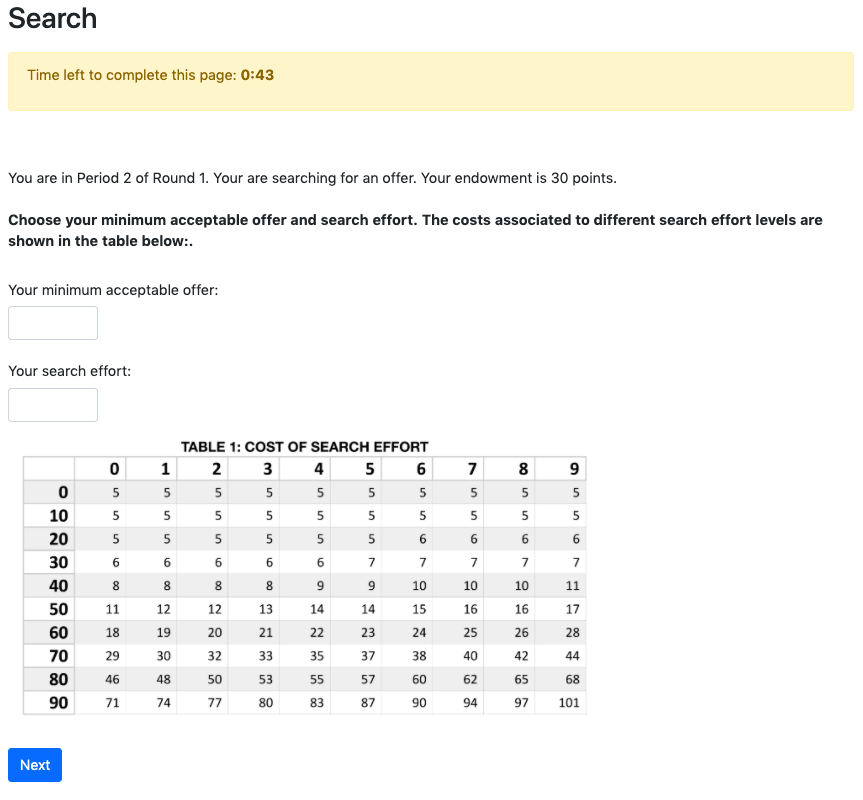


Figure 1: Screenshot of the decision screen

Once you submit your minimum acceptable offer and search effort, the computer randomly determines using your search effort level whether you receive an offer. If you receive an offer, the offer will be randomly selected between 1 and 100 Points, with every integer value having the same chance to be selected.

Then, there are **three** possible outcomes:

1. **You do not receive an offer.** Then, with 95% chance the Round continues to the next Period and you will search again. You will be asked to make new decisions again. With 5% chance the Round ends here.
2. **You receive an unacceptable offer:** it is below you minimum acceptable offer. Then, with 95% chance the Round continues to the next Period and you will search again. You will be asked to make new decisions again. With 5% chance the Round ends here.
3. **You receive an acceptable offer:** it is above or equal to your minimum acceptable offer. Then, you accept this offer and your search is over. There are no other decisions to be made in this Round. Your payoff will be equal to the value of the offer for the remaining Periods of this Round. The number of remaining Periods is randomly determined based on the following rule: after each Period, there is a 95% chance that the Round continues to the next Period and a 5% chance that the Round ends.

You will be informed which of these three outcomes is realized. You will see on the screen for 40 seconds:

- whether you have received an offer,
- if yes, what the value of the offer was, and whether or not the value was at least as large as your minimum acceptable offer,
- your chosen search effort,
- your chosen minimum acceptable offer,
- the search costs you paid,
- your Points earned from the Period.

This process is repeated until the Round comes to an end. Your total payoff from this Round will be computed as the **sum of your Points from all Periods** in this Round. The following examples illustrate how your total payoff from a Round is calculated. The numbers in these examples have been chosen for illustration only.

**Example 3:**

| Period | **1** | **2** | **3** | **4** | **5** | **6** | **7** | **8** | **9** |
| --- | --- | --- | --- | --- | --- | --- | --- | --- | --- |
| Search effort | 30 | 43 | 56 | 63 | 65 |  |  |  |  |
| Search cost | 6 | 8 | 15 | 21 | 23 |  |  |  |  |
| Payoff | 24 | 22 | 15 | 9 | 7 | 73 | 73 | 73 | 73 |

In this example, the Round lasted for 9 Periods. The participant searched in the first 5 Periods. In each of these Periods his/her income is equal to 30 minus the search costs. He/she accepted an offer in Period 5, the value of the offer is 73. He/she receives this amount from Period 6 on until the end of the Round in Period 9. The total payoffs from this Round are 24 + 22 + 15 + 9 + 7 + 4x73 = **369**.

**Example 4:**

| Period | **1** | **2** | **3** | **4** | **5** | **6** | **7** | **8** | **9** | … | **23** |
| --- | --- | --- | --- | --- | --- | --- | --- | --- | --- | --- | --- |
| Search effort | 73 | 53 | 62 | 40 | 73 | 62 | 78 | 72 |  |  |  |
| Search cost | 33 | 13 | 20 | 8 | 33 | 20 | 42 | 32 |  |  |  |
| Payoff | -3 | 17 | 10 | 22 | -3 | 10 | -12 | -2 | 65 | 65 | 65 |

In this example, the Round lasted for 23 Periods. The participant searched in the first 8 Periods. In each of these Periods his/her income is equal to 30 minus the search costs. He/she accepted an offer in Period 8, the value of the offer is 65. He/she receives this amount from Period 9 on until the end of the Round in Period 23. The total payoffs from this Round are
-3 + 17 + 10 + 22 + (-3) + 10 + (-12) + (-2) + 15x65 = **1014**.

When the Round ends, you will see on the screen for 25 seconds:

- whether you have accepted an offer in this Round,
- if yes, the value of the offer, and the Period in which you accepted it,
- your total payoff from this Round,
- the number of Periods in this Round.

Part 1 of the Experiment consists of **8 Rounds** that has the same structure as described above. At the beginning of each Round, you start searching for an offer again. After the last Round ends, you will see in your screen which of the 8 Rounds has been randomly selected for payment. The total number of Points you earned in that Round will be your payoff from Part 1 of the experiment.

Part 2 of the experiment consists of a questionnaire. Please, answer all the questions. Some of the question will allow you to earn additional Points as it will be explained on the screen.

In the end of the Experiment, your total number of Points from Part 1 and Part 2 of the experiment will be converted to GBP at the rate:

**500 Points = 1 GBP**

This amount plus your show-up fee of 2.5 GBP will be paid to you privately.

Before we start the experiment, you will be asked to answer a set of questions to make sure that you understood the instructions.

**Experimental Instructions – Nudge1, Nudge2, Nudge1+2**

**Welcome and thank you for participating in this study.**

**Please, provide your Prolific ID:**

For participating in this experiment you will earn a **fixed show-up fee of 2.5 GBP.** You will earn **additional money** depending on your decisions. This additional amount will paid to you as **Bonus payment.**

**Please, read these instructions carefully so that you fully understand the decisions you need to make.**

**Part 1** of the experiment consists of **8 Rounds**. In each Round you can earn Points. At the end of the experiment, **one Round will be randomly selected** and the Points earned in that Round will determine your additional payment.

In **Part 2** of the experiment you will be asked to fill out a questionnaire, some of the questions will allow you to earn additional Points.

At the end of the experiment, **the Points you earned in Part 1 and Part 2 of the experiment will be converted to GBP** using the exchange rate:

**500 Points = 1 GBP**

This amount plus your show-up fee of 2.5 GBP will be paid to you privately.

**Description of a Round**

Each of the 8 Rounds of the experiment has the same structure. A Round consists of multiple Periods. The **number of Periods** within a Round is not fixed, but it is **randomly determined** by the computer using the following rule: after each Period, there is a 95% chance that the Round continues to the next Period and a 5% chance that the Round ends with the current Period. **Your total payoff from a Round will be the sum of Points you earned over all Periods in the Round.**

You start each Round by searching for offers. Offers are randomly drawn from the set of integer numbers between 1 and 100 Points (1,2,3,…100 Points), with every offer being **equally** **likely**. The value of the offer is important because once you accept an offer, you will earn the value of the offer for the remaining Periods in that Round.

In each Period while you are searching for an offer, you will make **the following** **decisions:**

Firstly, you choose your **minimum acceptable offer,** that is, the lowest offer that you are willing to accept. For example, if your minimum acceptable offer is 59 Points, you will *accept any offer above or equal to 59*, and you will *reject any offer below* 59 Points. Similarly, if your minimum acceptable offer is 79 Points, you will accept any offer above or equal to 79, and you will reject any offer below 79 Points.

Secondly, you decide your **search effort:** it determines the chance that you receive an offer, the higher your search effort is, the larger the chance that you receive an offer. You choose an integer number between 1 and 100. For example, if your search effort is 25, there is a 25% chance that you receive an offer in this Period, and a 75% chance that you do not receive an offer. Or if your search effort is 69, there is a 69% chance that you receive an offer in this Period, and a 31% chance that you do not receive an offer.

A higher search effort is more **costly**. The cost corresponding to all possible search effort levels is shown in Table 1. You will receive **an endowment of 30 Points** for each Period when you search. The cost associated to your search effort level will be deducted from your endowment. Your earnings from this Period will be 30 minus the cost of your search effort. Note that earnings can be negative in a particular Period.

**Example 1:**

The participant chooses the search effort to be equal to 37. The cost of this search effort level can be found in the row starting with ’30’ and the column with the header ‘7’ in Table 1. This shows that the cost is equal to 7.
The participant’s payoff from this Period is 30 - 7 = 23 Points.

**Example 2:**

The participant chooses the search effort to be equal to 75. The cost of this search effort level can be found in the row starting with ’70’ and the column with the header ‘5’ in Table 1. This shows that the cost is equal to 37.
The participant’s payoff from this Period is 30 - 37 = -7 Points.

**TABLE 1: COST OF SEARCH EFFORT**

|  | **0** | **1** | **2** | **3** | **4** | **5** | **6** | **7** | **8** | **9** |
| --- | --- | --- | --- | --- | --- | --- | --- | --- | --- | --- |
| **0** | 5 | 5 | 5 | 5 | 5 | 5 | 5 | 5 | 5 | 5 |
| **10** | 5 | 5 | 5 | 5 | 5 | 5 | 5 | 5 | 5 | 5 |
| **20** | 5 | 5 | 5 | 5 | 5 | 5 | 6 | 6 | 6 | 6 |
| **30** | 6 | 6 | 6 | 6 | 6 | 7 | 7 | 7 | 7 | 7 |
| **40** | 8 | 8 | 8 | 8 | 9 | 9 | 10 | 10 | 10 | 11 |
| **50** | 11 | 12 | 12 | 13 | 14 | 14 | 15 | 16 | 16 | 17 |
| **60** | 18 | 19 | 20 | 21 | 22 | 23 | 24 | 25 | 26 | 28 |
| **70** | 29 | 30 | 32 | 33 | 35 | 37 | 38 | 40 | 42 | 44 |
| **80** | 46 | 48 | 50 | 53 | 55 | 57 | 60 | 62 | 65 | 68 |
| **90** | 71 | 74 | 77 | 80 | 83 | 87 | 90 | 94 | 97 | 101 |

Figure 1 shows a screenshot of the decision screen. You will have 50 seconds to submit your decisions. If you finish earlier, you can click ‘Next’. If you do not submit your decisions within 50 seconds, you have no chance to receive an offer in this Period.


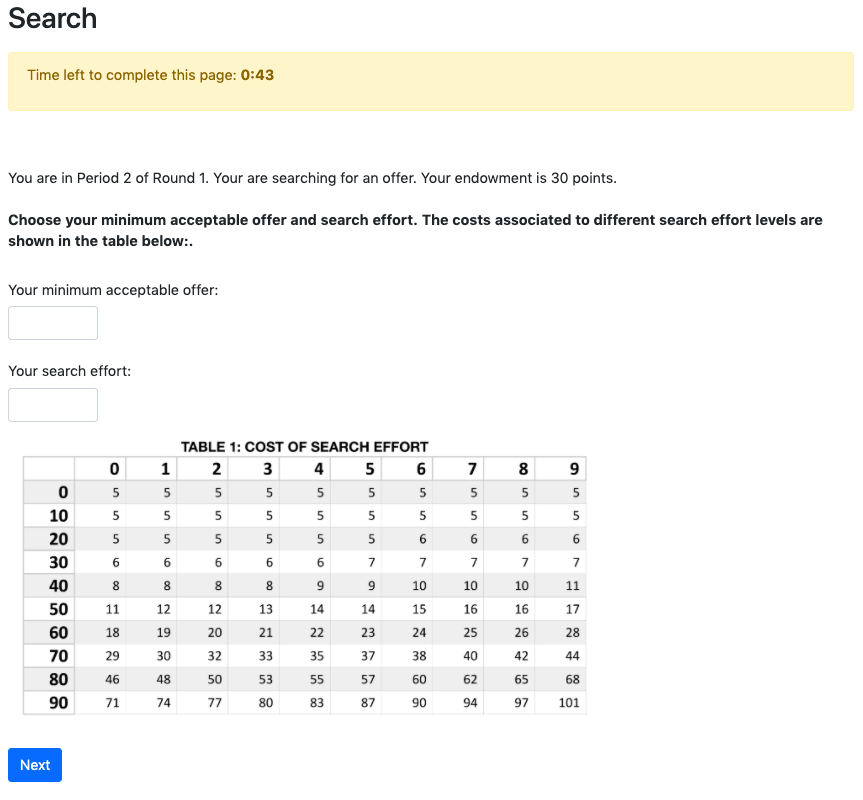


Figure 1: Screenshot of the decision screen

*Note: A message (titled ‘Note’) will appear on your decision screen. When making your choices on search effort and reservation wage, you are completely free to follow, or not, this message. The note will appear above the boxes where you need to insert your decisions.*

Once you submit your minimum acceptable offer and search effort, the computer randomly determines using your search effort level whether you receive an offer. If you receive an offer, the offer will be randomly selected between 1 and 100 Points, with every integer value having the same chance to be selected.

Then, there are **three** possible outcomes:

1. **You do not receive an offer.** Then, with 95% chance the Round continues to the next Period and you will search again. You will be asked to make new decisions again. With 5% chance the Round ends here.
2. **You receive an unacceptable offer: it is below you minimum acceptable offer.** Then, with 95% chance the Round continues to the next Period and you will search again. You will be asked to make new decisions again. With 5% chance the Round ends here.
3. **You receive an acceptable offer: it is above or equal to your minimum acceptable offer.** Then, you accept this offer and your search is over. There are no other decisions to be made in this Round. Your payoff will be equal to the value of the offer for the remaining Periods of this Round. The number of remaining Periods is randomly determined based on the following rule: after each Period, there is a 95% chance that the Round continues to the next Period and a 5% chance that the Round ends.

You will be informed which of these three outcomes is realized. You will see on the screen for 40 seconds:

- whether you have received an offer,
- if yes, what the value of the offer was, and whether or not the value was at least as large as your minimum acceptable offer,
- your chosen search effort,
- your chosen minimum acceptable offer,
- the search costs you paid,
- your Points earned from the Period.

This process is repeated until the Round comes to an end. Your total payoff from this Round will be computed as the **sum of your Points from all Periods** in this Round. The following examples illustrate how your total payoff from a Round is calculated. The numbers in these examples have been chosen for illustration only.

**Example 3:**

| Period | **1** | **2** | **3** | **4** | **5** | **6** | **7** | **8** | **9** |
| --- | --- | --- | --- | --- | --- | --- | --- | --- | --- |
| Search effort | 30 | 43 | 56 | 63 | 65 |  |  |  |  |
| Search cost | 6 | 8 | 15 | 21 | 23 |  |  |  |  |
| Payoff | 24 | 22 | 15 | 9 | 7 | 73 | 73 | 73 | 73 |

In this example, the Round lasted for 9 Periods. The participant searched in the first 5 Periods. In each of these Periods his/her income is equal to 30 minus the search costs. He/she accepted an offer in Period 5, the value of the offer is 73. He/she receives this amount from Period 6 on until the end of the Round in Period 9. The total payoffs from this Round are 24 + 22 + 15 + 9 + 7 + 4x73 = **369**.

**Example 4:**

| Period | **1** | **2** | **3** | **4** | **5** | **6** | **7** | **8** | **9** | … | **23** |
| --- | --- | --- | --- | --- | --- | --- | --- | --- | --- | --- | --- |
| Search effort | 73 | 53 | 62 | 40 | 73 | 62 | 78 | 72 |  |  |  |
| Search cost | 33 | 13 | 20 | 8 | 33 | 20 | 42 | 32 |  |  |  |
| Payoff | -3 | 17 | 10 | 22 | -3 | 10 | -12 | -2 | 65 | 65 | 65 |

In this example, the Round lasted for 23 Periods. The participant searched in the first 8 Periods. In each of these Periods his/her income is equal to 30 minus the search costs. He/she accepted an offer in Period 8, the value of the offer is 65. He/she receives this amount from Period 9 on until the end of the Round in Period 23. The total payoffs from this Round are
-3 + 17 + 10 + 22 + (-3) + 10 + (-12) + (-2) + 15x65 = **1014**.

When the Round ends, you will see on the screen for 25 seconds:

- whether you have accepted an offer in this Round,
- if yes, the value of the offer, and the Period in which you accepted it,
- your total payoff from this Round,
- the number of Periods in this Round.

Part 1 of the Experiment consists of **8 Rounds** that has the same structure as described above. At the beginning of each Round, you start searching for an offer again. After the last Round ends, you will see in your screen which of the 8 Rounds has been randomly selected for payment. The total number of Points you earned in that Round will be your payoff from Part 1 of the experiment.

Part 2 of the experiment consists of a questionnaire. Please, answer all the questions. Some of the question will allow you to earn additional Points as it will be explained on the screen.

In the end of the Experiment, your total number of Points from Part 1 and Part 2 of the experiment will be converted to GBP at the rate:

**500 Points = 1 GBP**

This amount plus your show-up fee of 2.5 GBP will be paid to you privately.

Before we start the experiment, you will be asked to answer a set of questions to make sure that you understood the instructions.

**Experimental Instructions - LowCost**

**Welcome and thank you for participating in this study.**

**Please, provide your Prolific ID:**

For participating in this experiment you will earn a **fixed show-up fee of 2.5 GBP.** You will earn **additional money** depending on your decisions. This additional amount will paid to you as **Bonus payment.**

**Please, read these instructions carefully so that you fully understand the decisions you need to make.**

**Part 1** of the experiment consists of **8 Rounds**. In each Round you can earn Points. At the end of the experiment, **one Round will be randomly selected** and the Points earned in that Round will determine your additional payment.

In **Part 2** of the experiment you will be asked to fill out a questionnaire, some of the questions will allow you to earn additional Points.

At the end of the experiment, **the Points you earned in Part 1 and Part 2 of the experiment will be converted to GBP** using the exchange rate:

**500 Points = 1 GBP**

This amount plus your show-up fee of 2.5 GBP will be paid to you privately.

**Description of a Round**

Each of the 10 Rounds of the experiment has the same structure. A Round consists of multiple Periods. The **number of Periods** within a Round is not fixed, but it is **randomly determined** by the computer using the following rule: after each Period, there is a 95% chance that the Round continues to the next Period and a 5% chance that the Round ends with the current Period. **Your total payoff from a Round will be the sum of Points you earned over all Periods in the Round.**

You start each Round by searching for offers. Offers are randomly drawn from the set of integer numbers between 1 and 100 Points (1,2,3,…100 Points), with every offer being **equally** **likely**. The value of the offer is important because once you accept an offer, you will earn the value of the offer for the remaining Periods in that Round.

In each Period while you are searching for an offer, you will make **the following** **decisions:**

Firstly, you choose your **minimum acceptable offer,** that is, the lowest offer that you are willing to accept. For example, if your minimum acceptable offer is 59 Points, you will *accept any offer above or equal to 59*, and you will *reject any offer below* 59 Points. Similarly, if your minimum acceptable offer is 79 Points, you will accept any offer above or equal to 79, and you will reject any offer below 79 Points.

Secondly, you decide your **search effort:** it determines the chance that you receive an offer, the higher your search effort is, the larger the chance that you receive an offer. You choose an integer number between 1 and 100. For example, if your search effort is 25, there is a 25% chance that you receive an offer in this Period, and a 75% chance that you do not receive an offer. Or if your search effort is 69, there is a 69% chance that you receive an offer in this Period, and a 31% chance that you do not receive an offer.

A higher search effort is more **costly**. The cost corresponding to all possible search effort levels is shown in Table 1. You will receive **an endowment of 30 Points** for each Period when you search. The cost associated to your search effort level will be deducted from your endowment. Your earnings from this Period will be 30 minus the cost of your search effort. Note that earnings can be negative in a particular Period.

**Example 1:**

The participant chooses the search effort to be equal to 37. The cost of this search effort level can be found in the row starting with ’30’ and the column with the header ‘7’ in Table 1. This shows that the cost is equal to 6.
The participant’s payoff from this Period is 30 - 6 = 24 Points.

**Example 2:**

The participant chooses the search effort to be equal to 92. The cost of this search effort level can be found in the row starting with ’90’ and the column with the header ‘2’ in Table 1. This shows that the cost is equal to 32.
The participant’s payoff from this Period is 30 - 32 = -2 Points.

**TABLE 1: COST OF SEARCH EFFORT**

|  | **0** | **1** | **2** | **3** | **4** | **5** | **6** | **7** | **8** | **9** |
| --- | --- | --- | --- | --- | --- | --- | --- | --- | --- | --- |
| **0** | 5 | 5 | 5 | 5 | 5 | 5 | 5 | 5 | 5 | 5 |
| **10** | 5 | 5 | 5 | 5 | 5 | 5 | 5 | 5 | 5 | 5 |
| **20** | 5 | 5 | 5 | 5 | 5 | 5 | 5 | 5 | 5 | 5 |
| **30** | 5 | 5 | 5 | 5 | 6 | 6 | 6 | 6 | 6 | 6 |
| **40** | 6 | 6 | 6 | 6 | 6 | 7 | 7 | 7 | 7 | 7 |
| **50** | 7 | 8 | 8 | 8 | 8 | 8 | 9 | 9 | 9 | 10 |
| **60** | 10 | 10 | 11 | 11 | 11 | 12 | 12 | 13 | 13 | 14 |
| **70** | 14 | 15 | 15 | 16 | 16 | 17 | 18 | 18 | 19 | 20 |
| **80** | 20 | 21 | 22 | 23 | 24 | 25 | 26 | 26 | 27 | 29 |
| **90** | 30 | 31 | 32 | 33 | 34 | 36 | 37 | 38 | 40 | 41 |

Figure 1 shows a screenshot of the decision screen. You will have 50 seconds to submit your decisions. If you finish earlier, you can click ‘Next’. If you do not submit your decisions within 50 seconds, you have no chance to receive an offer in this Period.


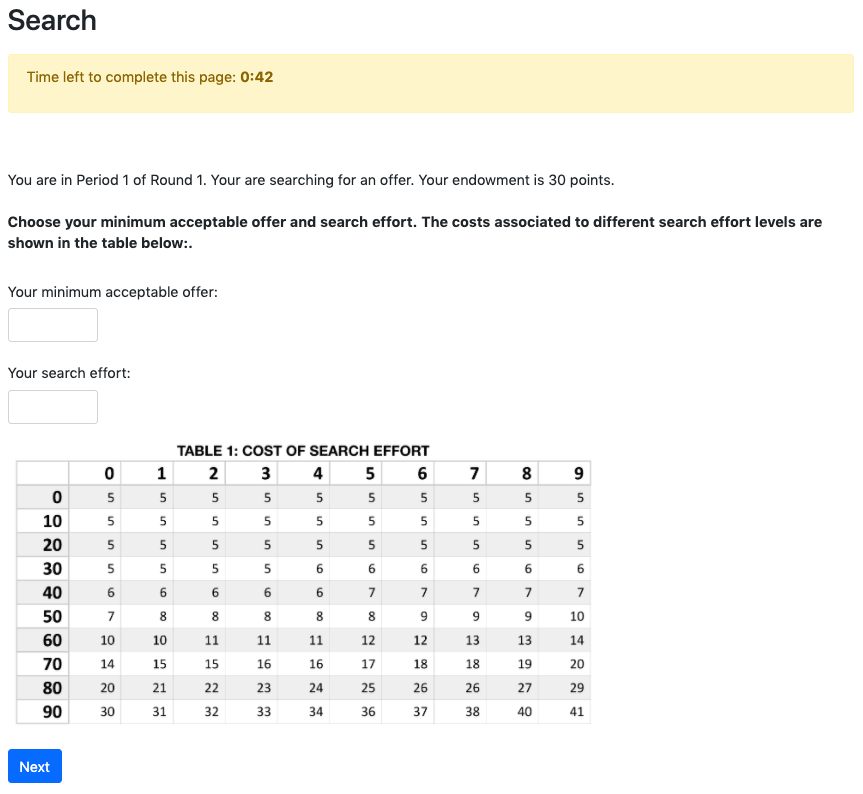


Figure 1: screenshot of the decision screen

Once you submit your minimum acceptable offer and search effort, the computer randomly determines using your search effort level whether you receive an offer. If you receive an offer, the offer will be randomly selected between 1 and 100 Points, with every integer value having the same chance to be selected.

Then, there are **three** possible outcomes:

1. **You do not receive an offer.** Then, with 95% chance the Round continues to the next Period and you will search again. You will be asked to make new decisions again. With 5% chance the Round ends here.
2. **You receive an unacceptable offer: it is below you minimum acceptable offer.** Then, with 95% chance the Round continues to the next Period and you will search again. You will be asked to make new decisions again. With 5% chance the Round ends here.
3. **You receive an acceptable offer: it is above or equal to your minimum acceptable offer.** Then, you accept this offer and your search is over. There are no other decisions to be made in this Round. Your payoff will be equal to the value of the offer for the remaining Periods of this Round. The number of remaining Periods is randomly determined based on the following rule: after each Period, there is a 95% chance that the Round continues to the next Period and a 5% chance that the Round ends.

You will be informed which of these three outcomes is realized. You will see on the screen for 40 seconds:

- whether you have received an offer,
- if yes, what the value of the offer was, and whether or not the value was at least as large as your minimum acceptable offer,
- your chosen search effort,
- your chosen minimum acceptable offer,
- the search costs you paid,
- your Points earned from the Period.

This process is repeated until the Round comes to an end. Your total payoff from this Round will be computed as the **sum of your Points from all Periods** in this Round. The following examples illustrate how your total payoff from a Round is calculated. The numbers in these examples have been chosen for illustration only.

**Example 3:**

| Period | **1** | **2** | **3** | **4** | **5** | **6** | **7** | **8** | **9** |
| --- | --- | --- | --- | --- | --- | --- | --- | --- | --- |
| Search effort | 30 | 43 | 56 | 63 | 65 |  |  |  |  |
| Search cost | 5 | 6 | 9 | 11 | 12 |  |  |  |  |
| Payoff | 25 | 24 | 21 | 19 | 18 | 73 | 73 | 73 | 73 |

In this example, the Round lasted for 9 Periods. The participant searched in the first 5 Periods. In each of these Periods his/her income is equal to 30 minus the search costs. He/she accepted an offer in Period 5, the value of the offer is 73. He/she receives this amount from Period 6 on until the end of the Round in Period 9. The total payoffs from this Round are 25 + 24 + 21 + 19 + 18 + 4x73 = **399**.

**Example 4:**

| Period | **1** | **2** | **3** | **4** | **5** | **6** | **7** | **8** | **9** | … | **23** |
| --- | --- | --- | --- | --- | --- | --- | --- | --- | --- | --- | --- |
| Search effort | 73 | 53 | 62 | 40 | 73 | 62 | 93 | 72 |  |  |  |
| Search cost | 16 | 8 | 11 | 6 | 16 | 11 | 33 | 15 |  |  |  |
| Payoff | 14 | 22 | 19 | 24 | 14 | 19 | -3 | 15 | 65 | 65 | 65 |

In this example, the Round lasted for 23 Periods. The participant searched in the first 8 Periods. In each of these Periods his/her income is equal to 30 minus the search costs. He/she accepted an offer in Period 8, the value of the offer is 65. He/she receives this amount from Period 9 on until the end of the Round in Period 23. The total payoffs from this Round are 14 + 22 + 19 + 24 + 14 + 19 + (-3) + 15 + 15x65 = **1099**.

When the Round ends, you will see on the screen for 25 seconds:

- whether you have accepted an offer in this Round,
- if yes, the value of the offer, and the Period in which you accepted it,
- your total payoff from this Round,
- the number of Periods in this Round.

Part 1 of the Experiment consists of **8 Rounds** that has the same structure as described above. At the beginning of each Round, you start searching for an offer again. After the last Round ends, you will see in your screen which of the 8 Rounds has been randomly selected for payment. The total number of Points you earned in that Round will be your payoff from Part 1 of the experiment.

Part 2 of the experiment consists of a questionnaire. Please, answer all the questions. Some of the question will allow you to earn additional Points as it will be explained on the screen.

In the end of the Experiment, your total number of Points from Part 1 and Part 2 of the experiment will be converted to GBP at the rate:

**500 Points = 1 GBP**

This amount plus your show-up fee of 2.5 GBP will be paid to you privately.

Before we start the experiment, you will be asked to answer a set of questions to make sure that you understood the instructions.
